# Use of an Alkaline Wastewater Stream to Increase the Initial pH of Whey and Recover a Microbial Biomass with High Protein Content

**DOI:** 10.3390/foods15112022

**Published:** 2026-06-04

**Authors:** Marisol Pérez-Cortés, Janet A. Gutiérrez-Uribe, Mariana Franco-Morgado

**Affiliations:** 1 Tecnologico de Monterrey, Escuela de Ingenieria y Ciencias, Atlixcáyotl 5718, Reserva Territorial Atlixcáyotl, Puebla 72453, Mexico; a00838175@tec.mx; 2 Tecnologico de Monterrey, Escuela de Ingenieria y Ciencias, Av. Eugenio Garza Sada 2501 Sur, Monterrey 64849, Mexico; jagu@tec.mx

**Keywords:** microalgae–cyanobacteria, phototrophic–mixotrophic cultivation, sweet whey, nejayote, proteins, bioconversion

## Abstract

Sweet whey (SW) and nejayote (NE), two agro-industrial wastewaters generated in Mexico, were evaluated as growth media for the cultivation of an alkaliphilic microalgae–cyanobacteria consortium (AMC) which has been reported to contain *Nannochloropsis* sp. and *Pseudanabaena* sp. at different initial pH (8, 9, and 10). Phototrophic-mixotrophic cultivation was conducted for 14 d using nejayote with biomass (NEB), sweet whey with biomass (SWB), and a mixture of nejayote and sweet whey with biomass (NESWB) to assess organic matter removal, biomass formation, and metabolite dynamics. The highest chemical oxygen demand (COD) removal was observed in NEB at pH 8, reaching 91.66% and a final COD of 1.04 g L^−1^. Initial pH values of 9 and 10 maintained alkaline conditions through phototrophic–mixotrophic cultivation, indicating stable biological pH regulation associated with photosynthetic activity. NESWB promoted higher biomass production, particularly at pH 9, suggesting enhanced conversion of organic matter into suspended solids. Moreover, the highest intracellular protein content (30.50 ± 0.90% dry weight) was obtained in NESWB at pH 10, supported by FTIR and SDS-PAGE analyses that indicated changes in protein-related spectral features and band profiles. Biomass reached 4.77 ± 0.80 g L^−1^ and COD decreased from 14.60 ± 0.70 to 4.18 ± 0.31 g L^−1^. These results demonstrate that the integration of sweet whey and nejayote under alkaline conditions enables simultaneous wastewater treatment and production of protein-rich biomass, highlighting a sustainable strategy for agro-industrial residue valorization.

## 1. Introduction

Biosource utilization has gained significant attention as industries seek sustainable approaches for waste management and resource recovery. Sweet whey (SW), generated after casein precipitation during cheesemaking, and nejayote (NE), the alkaline effluent from maize nixtamalization, both exhibit high nutrient concentrations and elevated organic loads. NE typically presents approximately 40 g O_2_ L^−1^ of chemical oxygen demand (COD), a pH near 11, high total suspended solids close to 8 g L^−1^, and elevated electrical conductivity (∼4500 µS cm^−1^) [1]. In terms of nutrients, NE has been reported to have a total phosphorus content (TP) of 83.1–178 mg P L^−1^ and a total nitrogen content (TN) of 95–440 mg N L^−1^ [2]. In contrast, SW presents a higher nutrient load with a COD of 50 to 70 g L^−1^, 1610 mg L^−1^ of TP, and 543 mg L^−1^ of TN [3]. On the other hand, SW usually has pH values between 6 and 7, and a high concentration of lactose (65.91 g L^−1^), proteins (3.80 g L^−1^), lipids (2.41 g L^−1^), and minerals [4,5]. The production of one kilogram of hard or semi-hard cheese yields around nine liters of whey released into the environment [6]. Along with the estimated annual release of 14.4 million m^3^ of NE, these effluents represent a significant environmental burden in Mexico [7]. Limited infrastructure in small-scale dairy and tortilla industries often leads to the direct release of untreated SW and NE into water bodies, promoting eutrophication and aquatic ecosystem degradation [2,8]. These conditions highlight the urgent need for low-cost and scalable biotechnological solutions, among which microalgae and cyanobacteria have emerged as promising candidates.

Microalgae and cyanobacteria are promising biological agents for bioprocesses and bioproduct formation, owing to their metabolic flexibility, rapid growth, and ability to assimilate nutrients under diverse and often extreme conditions [9,10]. These microorganisms have attracted increasing attention. They are valued not only for biomass production but also for the generation of high-value bioproducts, such as proteins, pigments, and carbohydrates [11]. Simultaneously, they contribute to reducing chemical oxygen demand (COD). For instance, Garza-Valverde et al. [12] demonstrated that *Scenedesmus acutus* and *Haematococcus pluvialis* cultivated in mixtures of nejayote and food waste leachate (with pH maintained between 8 and 10) achieved biomass concentrations of up to 5.34 g L^−1^ for *S. acutus* for 20 d, with COD reductions of up to 87%, along with ammonium (83%) and orthophosphate (84.1%) removal. Similarly, López-Pacheco et al. [9] reported that *Arthrospira maxima* and *Chlorella vulgaris* cultivated for 20 d in mixtures of nejayote and swine wastewater (with no pH adjustment; the culture pH remained between 8 and 10) showed enhanced biomass growth, reaching up to 1.70 g L^−1^ for *C. vulgaris*, while simultaneously achieving removals of up to 96% COD, 91% total nitrogen, and 85% total phosphorus. Furthermore, Del Valle-Real et al. [13] evaluated a consortium of alkaliphilic microalgae–cyanobacteria cultivated in nejayote (with no pH adjustment; the culture pH remained between 9.5 and 8.8), achieving COD reductions of up to 30% within 12 d, with a biomass protein content of 16.00 ± 1.00%. These results confirm that waste materials such as nejayote, whey, and mixed agro-industrial effluents can be effective substrates for cultivating microalgae and cyanobacteria.

In addition to microalgal systems, specific genera, such as *Nannochloropsis* and filamentous cyanobacteria (e.g., *Pseudanabaena* spp.), have demonstrated significant potential for wastewater treatment due to their complementary metabolic capabilities. *Nannochloropsis* species are known for their robust and efficient nutrient assimilation, with reported removal efficiencies of up to 59.85% for ammonium and 90.44% for phosphate [14]. Similarly, cyanobacteria such as *Pseudanabaena* have demonstrated high nitrogen removal efficiency, particularly in nutrient-rich effluents, achieving total nitrogen removals of up to 86.44% in dairy industry wastewater systems [15]. Microalgal-cyanobacterial consortia can outperform monocultures due to their functional complementarity, where different organisms contribute to nutrient uptake under diverse environmental conditions [16,17,18]. Furthermore, consortia exhibit greater resilience to environmental fluctuations and improve biomass recovery due to natural flocculation and aggregation phenomena [19,20]. However, the success of these bioprocesses heavily depends on physicochemical factors, especially pH, which influence nutrient availability and microbial activity.

pH influences nutrient solubility, carbon availability, enzymatic activity, membrane stability, and electron transport in microalgae and cyanobacteria [21]. Alkaline conditions (pH 8–10) have been shown to promote biomass accumulation, protein production, and nutrient assimilation in several strains. These processes occur through increased inorganic carbon availability, improved nitrogen uptake, and the stimulation of enzymatic pathways involved in protein biosynthesis [22,23]. Highly alkaline effluents, such as NE, inhibit the growth of undesirable microorganisms and favor alkaliphilic phototrophic communities, thus reducing microbial competition and limiting unwanted nutrient consumption [13]. Consequently, adjusting the initial pH is relevant to both microbial performance and process implementation, as alkaline operation has been associated with a lower risk of contamination and greater robustness in treating nutrient-rich waste streams [22]. In this context, the combination SW and NE is particularly relevant, as this strategy moderates extreme alkalinity while increasing the availability of organic sources of carbon and nitrogen, including lactose and soluble proteins, thus creating favorable physicochemical conditions for the availability and assimilation of nutrients in alkaline operation [2,24].

Therefore, this study evaluates the use of alkaline wastewater as a functional medium to mitigate the high organic load of sweet whey while simultaneously promoting protein-rich biomass production by a microalgae-cyanobacteria consortium. By combining sweet whey with nejayote, an inherently alkaline effluent, the approach exploits alkaline operating conditions to enhance chemical oxygen demand (COD) removal, stabilize culture pH, and favor the assimilation of organic carbon and nitrogen into cellular protein. The findings provide critical insights into pH-driven bioprocess performance and support the development of sustainable bioreaction-kinetics-based strategies for the valorization of agro-industrial residues through integrated wastewater treatment and biomass production in Mexico.

## 2. Materials and Methods

### 2.1. Substrates and Microbial Consortium

A local tortilla producer in Puebla, Mexico, provided nejayote (NE). It was left to settle for 3 h to remove suspended solids, and the supernatant was used for all experiments. Sweet whey (SW) was provided by a dairy processing plant located in Puebla, Mexico, and stored at 4 °C for a maximum of 18 h before processing. An alkaliphilic microalgae–cyanobacteria consortium (AMC) obtained from a soda lake (electrical conductivity of 12 mS cm^–1^ and pH between 9 and 11) located at Texcoco, Mexico, was used in all experiments. It is mainly composed of *Nannochloropsis* sp. and *Pseudanabaena* sp. [25] and grown in a mineral salt medium (MSM) composed of (g L^−1^) Na_2_CO_3_ (4.03), NaHCO_3_ (13.61), NaCl (1), K_2_HPO_4_ (1), K_2_SO_4_ (1), CaCl_2_·2H_2_0 (0.4), KNO_3_ (2.53), and MgCl_2_·6H_2_O (0.203) according to de los Cobos-Vasconcelos et al. [26].

### 2.2. Experimental Design and Cultivation Conditions

Phototrophic–mixotrophic cultivation was conducted in 1 L Erlenmeyer flasks incubated in an Algaetron chamber AG 130 (PSI, spol. s r. o., Drásov, Czech Republic) illuminated with a 12:12 h light/dark cycle at a photosynthetically active radiation (PAR) of around 150 µmol m^−2^s^−1^ at 25.00 ± 1.00 °C and agitated at 150 rpm for 14 d. The flasks were inoculated with AMC to achieve a total suspended solids (TSS) concentration of 3 g L^−1^ dry weight. NE, SW, and mixed nejayote-whey (NESW) were used as culture media. The NESW medium was prepared using equal volumes of NE and SW. The effect of initial pH (8, 9, and 10) was evaluated across three treatments: nejayote with biomass (NEB), sweet whey with biomass (SWB), and the mixture of nejayote and sweet whey with biomass (NESWB). The pH of the experiments described above was adjusted using 5 N HCl and 5 N NaOH, respectively. The pH was adjusted only at the beginning of the experiment to the desired values (8, 9, and 10) and was not actively controlled during cultivation, allowing it to vary according to biological activity and system dynamics. Control experiments (without biomass) were conducted for each culture medium (NE, SW, and NESW) under the same initial pH conditions (8, 9, and 10) and cultivation parameters. TSS values in control systems were reported as absolute concentrations, since these treatments were used to characterize background transformations occurring in uninoculated, non-sterile media. All control experiments were performed in triplicate and monitored in parallel with inoculated systems.

### 2.3. Monitoring of Cultivation Performance

pH, dissolved oxygen concentration (DO), conductivity, and oxidation-reduction potential (ORP) were measured in the culture medium (NE, SW, and NESW) and throughout the phototrophic-mixotrophic cultivation using a HI98194 multiparameter (HANNA Instruments, Nușfalău, Romania). While all parameters were continuously monitored, only pH and DO are reported in the Results Section, as they provided the most relevant information for interpreting system performance. Conductivity and ORP measurements were used for process assessment but are not shown.

The concentration of total suspended solids (TSS) was measured in the culture medium (NE, SW, and NESW) and throughout the phototrophic-mixotrophic cultivation according to standard methods [27] using pre-weighed glass fiber filters (Whatman GF/F, 0.7 µm pore size). A total of 5 mL of each sample was filtered, and the retained solids were dried at 105 °C until constant weight. To avoid overestimating biomass due to suspended solids in the substrates, TSS measurements were corrected by subtracting the initial TSS contribution of each culture medium (without inoculum). Therefore, the reported TSS values correspond to biomass-associated dry weight.

#### 2.3.1. Chemical Analyses of the Supernatant

The supernatant obtained after TSS filtration was used for chemical analysis. The chemical oxygen demand (COD) was analyzed spectrophotometrically with a DR6000 spectrophotometer (HACH, Düsseldorf, Germany) using a block digestor in the presence of dichromate at 150 °C according to O’Dell [28].

Total nitrogen (TN) and total phosphorus (TP) were determined using Hach digestion and analysis kits. TN was measured following the persulfate digestion method (Hach Method 10072), in which all nitrogen species were converted to nitrate prior to analysis. TP was determined using the molybdovanadate method after acid persulfate digestion (Hach Method 10127), where all phosphorus forms were converted to orthophosphate. The resulting orthophosphate reacts with molybdovanadate reagents to form a yellow-colored complex. All TN and TP measurements were performed spectrophotometrically using a DR6000 spectrophotometer (HACH, Düsseldorf, Germany).

Total soluble carbohydrate (TSC) was analyzed in the supernatant using a phenol–sulfuric acid reaction according to DuBois et al. [29].

Total soluble protein (TSP) concentration was analyzed in the supernatant using Folin–Ciocalteu reagent according to Lowry et al. [30].

Supernatant reducing sugar (RS) quantification was performed with a modified method according to Miller [31]. First, 300 µL of the supernatant sample to be analyzed was transferred to a microtube, and 300 µL of the DNS reagent was added (previously tempered for no more than 30 min). The tubes were then placed in a thermostatic bath at 95 °C for 5 min, and the reaction was stopped by immediately placing the tubes in an ice bath for an additional 5 min. After this time, 50 µL of the reaction solution was deposited in a well of a 96-well plate, and 250 µL of distilled water was added. The absorbance was determined at 540 nm.

#### 2.3.2. Biomass Macromolecular Composition

Biomass samples were collected at the beginning and at the end of the bioreaction kinetics period. For each sampling point, 50 mL of culture was centrifuged at 3600 rpm for 10 min at room temperature to separate the biomass from the culture medium. The resulting pellet was washed three times with distilled water to remove residual soluble compounds from the culture medium. The washed biomass was subsequently freeze-dried and stored until further biochemical analyses.

The carbohydrate content of the freeze-dried biomass was determined after acid hydrolysis, followed by quantification using the phenol-sulfuric acid method according to DuBois et al. [29], as calculated using Equation (1):

(1) $$Carbohydrate\;content\;\left(\%\right)=\frac{C\;\left(\frac{mg}{mL}\right)\times \;V\;(mL)}{m_{dry\;biomass}\left(mg\right)}\;\;\;$$

where *C* is the concentration of carbohydrates in the liquid extract, *V* is the total volume of the liquid extract, and *m_dry biomass_* is the starting dry mass of the biomass sample used for the extraction.

The protein content of the freeze-dried biomass was measured following alkaline hydrolysis, adapted from Pruvost et al. [32]. First, 20 mg of dried biomass was suspended in 4 mL of 1 M NaOH, vortexed, and heated at 95 °C for 20 min. After cooling, the solution was neutralized, centrifuged at 4000 rpm for 20 min, and the supernatant was used for protein determination via the Lowry method, as per Lowry et al. [30], and calculated with Equation (2):

(2) $$Protein\;content\;\left(\%\right)=\frac{C\;\left(\frac{mg}{mL}\right)\times V\;(mL)}{m_{dry\;biomass}\left(mg\right)}\;\;\;\;\;\;$$

where C is the concentration of protein in the liquid extract, V is the total volume of the liquid extract, and *m_dry biomass_* is the starting dry mass of the biomass sample used for the extraction.

The lipid content of the harvested biomass was determined following a modified chloroform-methanol extraction method based on Sirés and Brillas [33], following the procedure described by Lu et al. [34]. Approximately 40 mg of freeze-dried biomass was transferred into a glass tube and mixed with 2 mL of chloroform:methanol (2:1, *v/v*). Lipid extraction was assisted by ultrasonication using an ultrasonic bath (Branson 5510 Ultrasonic, Branson Ultrasonics, Danbury, CT, USA) for 15 min. After extraction, the mixture was centrifuged to separate the organic phase from the residual biomass. The extraction procedure was repeated three times to ensure complete lipid recovery. The combined organic extracts were concentrated using a rotary evaporator (HS-2005S-N Rotary Evaporator, Hahnshin Scientific, Gimpo-si, Republic of Korea) at 40 °C. The remaining lipid residue was gravimetrically quantified. Lipid content was expressed as a percentage of lipid mass relative to the dry biomass mass according to Equation (3):

(3) $$Lipid\;content\;\left(\%\right)=\;\frac{m_{lipid\;}(mg)}{m_{dry\;biomass}\;(mg)}\;\times \;100$$

where *m_lipid_* is the mass of extracted lipids and *m_dry biomass_* is the dry biomass used for the extraction.

Ash content was determined by gravimetric analysis. Approximately 40 mg of freeze-dried biomass was placed in previously weighed porcelain crucibles and incinerated in a muffle furnace (FE-340, Felisa, Jalisco, Mexico) at 550 °C for 12 h. After combustion, the crucibles were allowed to cool in a desiccator to room temperature and then weighed to determine the remaining inorganic residue. Ash content was expressed as the percentage of ash relative to the dry biomass mass according to Equation (4):

(4) $$Ash\;content\;\left(\%\right)=\frac{m_{ash}\;(mg)}{m_{dry\;biomass}\;(mg)}\times \;100\;\;\;\;$$

where *m_ash_* is the mass of ash remaining after combustion and *m_dry biomass_* corresponds to the initial dry biomass mass.

##### ATR-FTIR Spectral Characterization of Biomass

The presence of functional groups in the lyophilized fermented products (biomass) was analyzed using a Fourier transform infrared (FTIR) spectrophotometer (IRTracer-100, Shimadzu, Japan) equipped with an attenuated total reflection (ATR) accessory. Data were acquired from 4000 to 400 cm^−1^ with a resolution of 4 cm^−1^. Analysis of the obtained data and deconvolution of the second derivative of the amide I region (1600–1700 cm^−1^) were performed using Origin 2018 software (OriginLab, Northampton, MA, USA). Relative intensity variations were quantified by the percentage of the integrated area of each band. FTIR spectra were baseline-corrected and normalized to the amide I band prior to comparison. Readings for each sample were taken in triplicate.

##### SDS-PAGE Profiling of Intracellular Proteins

Intracellular proteins were extracted and separated by sodium dodecyl sulfate-polyacrylamide gel electrophoresis (SDS-PAGE) for molecular weight profiling. Freeze-dried biomass was resuspended in distilled water, and protein extracts were adjusted to a final concentration of 4 mg mL^−1^. A loading volume of 20 µL per lane was used. Prior to electrophoresis, samples were denatured by heating and combined with a reducing loading buffer containing Tris-HCl (0.5 M, pH 6.8), glycerol (20%), and SDS (4%). Protein separation was carried out using a discontinuous gel system consisting of a 5% stacking gel and a 10% resolving gel. Electrophoresis was performed under constant voltage conditions (120 V) until adequate band resolution was achieved. Following separation, gels were stained with Coomassie Brilliant Blue R-250 (0.1%, *w/v*) and subsequently destained to visualize protein bands [35]. Band patterns and apparent molecular weights were analyzed using ImageJ software version 1.5g (National Institutes of Health, Bethesda, MD, USA). Molecular weight estimation was performed by comparison with Precision Plus Protein™ All Blue Prestained Protein Standards covering a range of 10–250 kDa.

### 2.4. Statistical Analysis

Results were presented as the mean and standard deviation from three replicates of each biological experiment. Data were analyzed using a three-way analysis of variance (ANOVA), considering fermentation time (days 0–14), culture medium with biomass (NEB, SWB, and NESWB), and initial pH (8, 9, and 10) as fixed factors. Mean comparisons were conducted using Tukey´s honestly significant difference (HSD) test. Differences were regarded as statistically significant at *p* < 0.05. The data analysis was performed using JMP 18 (SAS Institute, Cary, NC, USA).

## 3. Results and Discussions

### 3.1. Monitoring of Cultivation Performance

#### 3.1.1. Culture Media

As expected, NE showed the highest pH (10.62 ± 0.08) and conductivity (6.50 ± 0.07 mS cm^−1^), and a lower COD (18.40 ± 0.17 g L^−1^) than whey (Table 1). These values were associated with residual calcium hydroxide from the nixtamalization process. Similar alkaline conditions and elevated ionic strength have been reported for nixtamalization wastewater [13,36]. In comparison, SW presented the lowest pH (5.65 ± 0.06) and the highest COD (71.00 ± 0.25 g L^−1^) (Table 1) as reported previously (60 to 80 g L^−1^) [37]. SW also had a higher total solids content (16.97 ± 2.11 g L^−1^) than NE (0.69 ± 0.16 g L^−1^). Previous studies have reported that SW exhibits acidic to near-neutral pH values (5–7) [6,38].

The physicochemical profile of NESW (9.20 ± 0.08; conductivity 5.84 ± 0.04 mS cm^−1^; COD 56.10 ± 0.08 g L^−1^; total suspended solids 9.98 ± 0.18 g L^−1^) reflected an intermediate condition between the highly alkaline NE and the highly organic-loaded SW (Table 1). Although studies specifically combining nejayote and whey are currently limited, previous co-treatment approaches using mixed-waste streams have shown that substrate blending can enhance biomass formation and intracellular metabolite accumulation compared to single-waste systems. Previous studies have demonstrated that blending waste streams and operating under mixotrophic conditions can substantially enhance both biomass production and intracellular metabolite accumulation. Zheng et al. [39] reported that *C. vulgaris* and mixing piggery and brewery wastewaters at an optimal C/N ratio of 7.9 resulted in biomass concentrations of up to 2.85 g L^−1^, together with high removal efficiencies for ammonium (100%), total nitrogen (96%), total phosphorus (90%), and COD (93%). Similarly, Rubiyatno et al. [40] showed that mixotrophic cultivation of *Euglena gracilis* using sewage effluent supplemented with organic waste streams achieved paramylon contents as high as 67.70% (*w/w*) and paramylon productivities of up to 47.80 mg L^−1^ d^−1^, highlighting the strong potential of mixed-waste-derived substrates to promote intracellular metabolite accumulation under mixotrophic operation.

#### 3.1.2. Phototrophic-Mixotrophic Cultivation

##### pH Evolution and Associated Process Performance at Initial pH 8

In the NEB treatment, pH increased significantly at 2, 4, and 6 d (8.79 ± 0.10, 8.61 ± 0.20, and 8.74 ± 0.00, respectively) compared to the initial value of 8.04 ± 0.40 (Figure S1). This increase in pH was consistent with an initial predominance of photosynthetic processes, which promote net proton uptake and alkalinization of the medium. A similar behavior has been reported in microalgae cultivated in food-processing wastewater, where enhanced photosynthetic activity during early growth stages increases inorganic carbon uptake, resulting in a progressive increase in culture pH [41]. This was supported by the increase in dissolved oxygen (Figure S2) from 27.90 ± 1.27% on day 0 to 61.33 ± 1.27% on day 6. Subsequently, a decrease was observed on day 8 to a pH of 6.20 ± 0.20. The subsequent pH decline reflects increased respiratory and heterotrophic activity, resulting in CO_2_ hydration to carbonic acid (H_2_CO_3_) and net proton release [42]. Similar patterns have been described in mixed and wastewater-based microalgae cultures, where the balance between photosynthetic carbon fixation and the release of acidic metabolites governs the evolution of net pH [10]. In this context, Abiusi et al. [43] investigated an oxygen-balanced mixotrophic microalgal system under controlled light availability and carbon supply, demonstrating that enhanced photosynthetic activity promotes CO_2_ fixation and proton uptake, leading to medium alkalinization, whereas increased respiratory metabolism results in transient acidification through CO_2_ release and carbonic acid formation.

Regarding NESWB treatment, a decrease in pH was exhibited, reaching 6.54 ± 0.02 on day 2. No significant differences were observed on days 6 (6.54 ± 0.01) and 8 (6.45 ± 0.05) relative to day 2. A subsequent increase was detected on day 10 (pH 8.01 ± 0.17), followed by a significant rise to 8.38 ± 0.07 on day 14. The SWB treatment showed a pH profile like that of NESWB; pH decreased on day 2 (6.18 ± 0.00) and remained statistically unchanged on days 4 and 6. An increase occurred on day 8 (pH 8.71 ± 0.02), and by day 10 it had increased to 8.97 ± 0.00. No significant differences were observed between days 10, 12 (8.95 ± 0.04), and 14 (9.07 ± 0.00). The shift toward alkaline pH conditions observed in the NESWB and SWB treatments after the initial acidification phase suggests a transition toward enhanced photosynthetic activity of the consortium. This trend was supported by the increase in DO (Figure S2), which in NESWB increased from 25.20 ± 2.22% on day 0 to 58.45 ± 1.27% on day 14, while in SWB, DO increased from 29.20 ± 0.20% to 36.76 ± 0.81% over the same period.

At an initial pH of 8, the pH evolution in the uninoculated controls was highly dependent on the medium. Pronounced early acidification was observed only in the whey-containing media. In NESW, both systems gradually recovered to near-neutral pH values by 14 d. In contrast, NE remained at near-neutral or slightly alkaline pH values throughout the culture period. These results indicate that early acidification at an initial pH of 8 was primarily associated with whey-containing substrates, while the nejayote matrix provided greater buffering capacity against pH changes.

##### pH Evolution and Associated Process Performance at Initial pH 9 and 10

The evolution of pH and its relationship with dissolved oxygen (DO) revealed distinct system behaviors under initial pH conditions of 9 and 10 (Figures S1 and S2). Cultures initiated at pH 9 exhibited more pronounced fluctuations compared to those initiated at pH 10. In NEB, the pH increased during the early cultivation phase (days 2–4), followed by a gradual decline toward day 14, while DO remained relatively stable, indicating a balanced interaction between photosynthetic and respiratory processes. In contrast, NESWB and SWB showed dynamic pH patterns characterized by alternating increases and decreases, which were accompanied by significant variations in DO. These patterns suggest shifts between net photosynthetic activity and respiratory demand, particularly evident in the sharp decline in DO at later stages of cultivation.

In comparison, cultures initiated at pH 10 maintained predominantly alkaline conditions with smaller pH variations across all treatments. Both NEB and NESWB exhibited gradual pH stabilization after the initial cultivation period, accompanied by sustained increases in DO, indicating continuous photosynthetic activity. Similarly, SWB showed an initial pH decrease followed by recovery and stabilization, while DO increased steadily throughout the cultivation period. These trends contrast with the more variable behavior observed at initial pH 9, suggesting that higher initial pH conditions promote a more stable physicochemical environment.

The persistence of alkaline conditions in cultures initiated at pH 9 and 10, despite gradual decreases over time, reflects the influence of photosynthetic CO_2_ assimilation on carbonate equilibrium. During active phototrophic/mixotrophic cultivation, CO_2_ uptake by microalgae shifts the system toward alkalinity, while oxygen production contributes to the oxidation of organic compounds. This coupling between pH and DO dynamics has been widely reported in microalgal systems treating wastewater, where photosynthetic activity enhances both alkalinity and oxygen availability, thereby supporting the removal of organic matter and nutrients [35,36].

In control NE at initial pH 9 and 10, the pH remained relatively stable, with only slight fluctuations around the initial value throughout the cultivation period. In contrast, the control NESW and SW exhibited a decrease toward acidic pH values. A distinction was observed between uninoculated controls and AMC-inoculated systems at initial pH 9 and 10. While control systems exhibited medium-dependent pH variations, including significant acidification in whey-containing media, inoculated cultures maintained relatively stable alkaline conditions across all treatments. This contrast indicates that pH stability in inoculated systems was not an inherent property of the culture media but rather a result of the metabolic activity of the microalgae–cyanobacteria consortium. In particular, maintaining an alkaline pH is consistent with photosynthetic CO_2_ uptake, which shifts the carbonate equilibrium and counteracts acidification observed in non-sterile controls. Therefore, the consortium contributed not only to substrate transformation but also to stabilizing the physicochemical environment.

##### Total Suspended Solids Performance and the Effect of Different Initial pH

**Figure 1:**
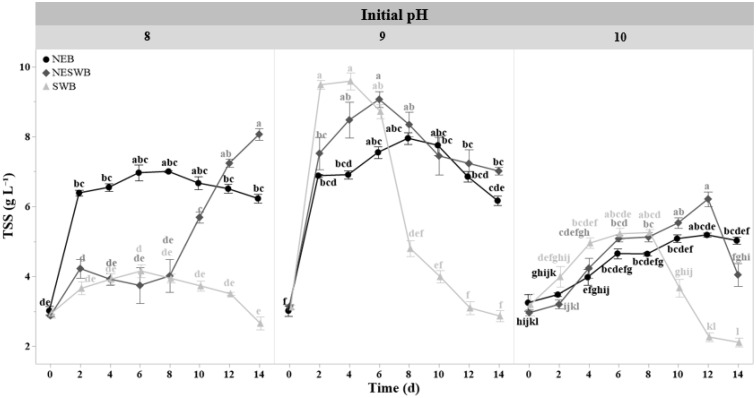
Changes in total suspended solids (TSS) observed during 14 d phototrophic–mixotrophic cultivation at an initial pH of 8, 9, and 10 of nejayote with biomass (NEB), sweet whey with biomass (SWB), and the mixture of nejayote and sweet whey with biomass (NESWB), all inoculated with an alkaliphilic microalgae–cyanobacteria consortium (AMC). TSS is represented by: 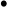 NEB, 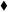 NESWB, 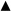 SWB. Values are means of triplicate. Values in each panel without letters in common are significantly different (*p* < 0.05).

Regarding TSS (Figure 1), it was observed that NESWB (days 4, 6, and 8), SWB (days 2, 4, and 6), and NEB (days 6, 8, and 10) at pH 9 had the highest concentration (9.58 ± 0.42 to 7.75 ± 0.49 g L^−1^). This indicates that a pH of 9 favored biomass growth. These results were consistent with findings by Garza-Valverde et al. [12], who reported that combining nejayote with food waste leachate enhanced the growth of *H. pluvialis*, highlighting the potential of mixed agro-industrial residues as effective culture media. Similarly, Cunha et al. [44] demonstrated that *Anabaena variabilis* efficiently consumed diverse organic carbon sources, including glucose, fructose, galactose, and lactose, and achieved significant biomass increases under mixotrophic conditions. These results confirm that the availability and diversity of organic carbon in the culture medium, rather than the concentration of a single compound, play a key role in converting organic matter into biomass.

In the SWB treatment, the final TSS concentration at 14 d was significantly lower across all initial pH conditions (pH 8, 2.65 ± 0.31 g L^−1^; pH 9, 2.86 ± 0.27 g L^−1^; and pH 10, 2.11 ± 0.21 g L^−1^) compared with NEB and NESWB. Regardless of the decrease in TSS, COD decreased significantly. This is explained by the fact that microalgae remove contaminants not only through cell growth but also through cell membrane adsorption and cell maintenance processes without cell growth. Consistent with this, previous studies on microalgal cultivation in dairy effluents have shown that efficient organic matter removal does not necessarily lead to increased biomass production. In this sense, Labbé et al. [45] reported that dairy wastewater supports microalgal activity and contaminant removal, but the high organic loads and the predominance of lactose impose physiological constraints, limiting cell growth. Similarly, Khalaji et al. [46] observed that while *C. vulgaris* removed organic matter from dairy wastewater, biomass production was influenced by wastewater dilution and nutrient balance. These observations are supported by the findings of Stratigakis et al. [47], who demonstrated that *Chlorella* growth in whey was influenced by physiological adaptability. Although the *Chlorella* strain remained metabolically active, biomass accumulation was limited under certain conditions, and a fraction of the assimilated carbon was redirected toward the production of extracellular polymeric substances.

The NESWB treatment at an initial pH of 8 showed an increase in biomass between days 8 and 14, consistent with an exponential (log) growth phase (Figure 1). This indicates effective physiological acclimatization of the AMC to the combined substrate. Youssef et al. [48] reported that the presence of whey as an organic carbon source can promote stable logarithmic growth once the cells adapt to mixotrophic conditions. The authors attributed this behavior to improved carbon availability and metabolic flexibility under moderate alkaline pH conditions, which favors the coordinated use of inorganic and organic carbon sources.

A distinction between inoculated and control cultivation (Table S1) is that TSS in the AMC treatments was reported as net accumulation, whereas in the control systems, it was reported as absolute values. Control experiments revealed that TSS changes occurred even in the absence of AMC, indicating that suspended solids were dynamically affected by physicochemical processes and by the possible activity of native microorganisms in the non-sterile medium. Therefore, TSS in the inoculated systems should be interpreted as biomass-associated suspended solids that develop on top of these background transformations. The use of net accumulation allows differentiation between the intrinsic effects of the medium and the net accumulation of solids attributable to microbial growth. In this context, the higher TSS values in the inoculated treatments reflect greater formation of biomass-associated solids rather than a direct comparison with the absolute TSS values of the control systems.

##### Chemical Analyses of the Supernatant

**Figure 2:**
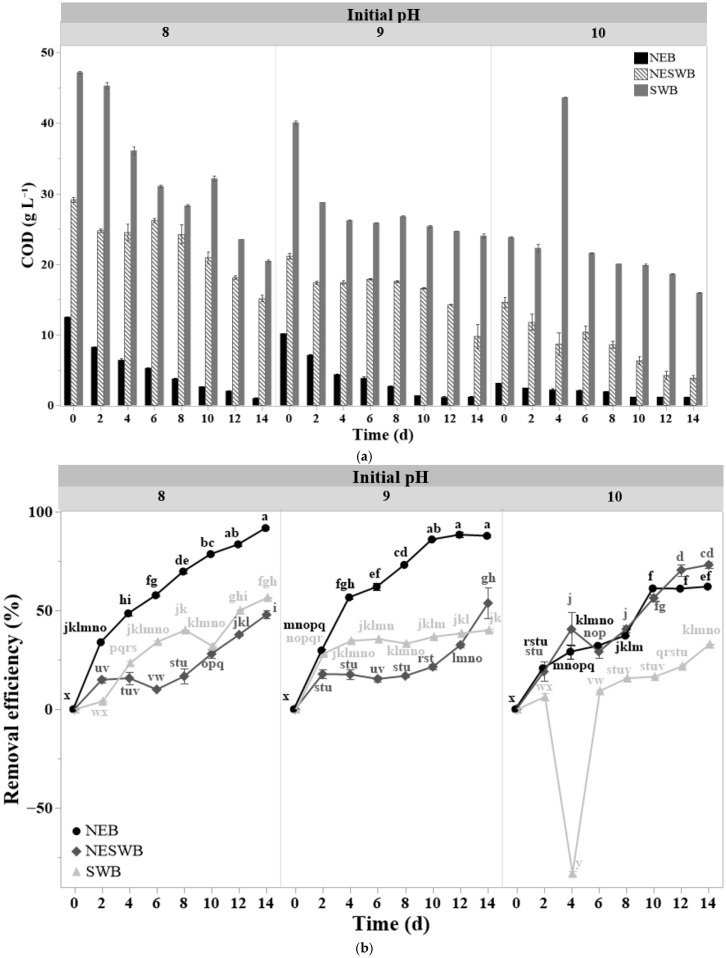
(**a**) Changes in chemical oxygen demand (COD) and (**b**) removal efficiency (%) observed during 14 d phototrophic–mixotrophic cultivation at an initial pH of 8, 9, and 10 of nejayote with biomass (NEB), sweet whey with biomass (SWB), and the mixture of nejayote and sweet whey with biomass (NESWB), all inoculated with an alkaliphilic microalgae-cyanobacteria consortium (AMC). COD is represented by: 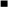 NEB, 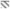 NESWB, 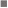 SWB. Removal efficiency (%) is represented by: 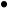 NEB, 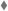 NESWB, 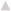 SWB. Values are means of triplicate. Values in each panel without letters in common are significantly different (*p* < 0.05).

Immediately after AMC inoculation, a reduction in COD was observed (Figure 2a). For NEB, the concentration decreased from 18.40 ± 0.17 g L^−1^ to 12.54 ± 0.02 g L^−1^ at pH 8, to 10.20 ± 0.01 g L^−1^ at pH 9, and to 3.18 ± 0.01 g L^−1^ at pH 10. Similarly, NESWB decreased from 56.10 ± 0.08 g L^−1^ to 29.12 ± 0.36, 21.18 ± 0.37, and 14.61 ± 0.70 g L^−1^ at pH 8, 9, and 10, respectively. In the case of SWB, concentrations declined from 71.00 ± 0.25 g L^−1^ to 47.20 ± 0.14 g L^−1^ at pH 8, 40.09 ± 0.22 g L^−1^ at pH 9, and 23.81 ± 0.10 g L^−1^ at pH 10. This decrease in COD following inoculation was consistent with the behavior reported by Zayas-Olivares et al. [49], due to dilution from inoculum incorporation and the precipitation of organic compounds by pH.

The highest removal efficiency of COD was recorded in the NEB treatment at pH 8 on day 14 with 91.66% (Figure 2b), reducing to 1.04 g L^−1^ (Figure 2a). This concentration was not significantly different from that obtained for the NEB treatment at an initial pH of 9 after 10 days, indicating that at initial pH 8 and 9, comparable organic matter removal efficiencies can be achieved. Notably, this behavior is consistent with the nutrient removal trends reported in Table 2, where the same treatment exhibited the highest removal efficiencies for total nitrogen (TN, 93.56 ± 2.22%) and total phosphorus (TP, 90.89 ± 4.42%). Similarly, the NESWB treatment at initial pH 10 re-established these parameters despite the high initial organic load, achieving a TN removal efficiency of 96.44 ± 1.03% and maintaining a COD removal above 70%, coinciding with a sustained increase in biomass (TSS). NESWB at initial pH 10 highlights the role of mixotrophy. The addition of whey provides the organic carbon necessary for the consortium, emulating the behavior of cyanobacteria such as *Pseudanabaena galeata* in dairy effluents, to carry out a reduction in nutrients that does not depend solely on precipitation but on an active biological assimilation driven by the substrate [15], achieving purification levels comparable to agro-industrial effluent treatment systems [9,50]. This trend has been reported in studies such as those by Valizadeh & Davarpanah [51], who highlighted that this pH range favors enzymatic activity and the solubilization of complex organic compounds, and Caporgno et al. [52], who reported that *Nannochloropsis oculata* can achieve phosphorus removals exceeding 96% in wastewater when physicochemical conditions favor cellular metabolism. In this case, the increase in TSS acts as a reliable indicator that nitrogen and phosphorus are being effectively incorporated into the synthesis of new proteins and cellular structures. The control cultivation showed statistically significant variations in COD over time (Table S1). However, these changes did not follow a constant decreasing trend. For example, in NESW at initial pH 10, COD decreased from 35.27 g L^−1^ to 25.10 g L^−1^ by day 8, but subsequently increased to 32.93 g L^−1^ by day 14. Similar fluctuation patterns were observed at pH 9 and 8, where COD initially increased or decreased but did not show sustained removal. These results suggest that COD variations in control systems were primarily associated with physicochemical processes such as particulate settling, solubilization of organic matter, or analytical variability, rather than true degradation. Despite the COD reduction achieved, the final concentration remained above the regulatory discharge limit of 0.21 g L^−1^ established by NOM-001-SEMARNAT-2021 for wastewater discharges [53].

This behavior indicates that COD reduction in microalgal systems is not exclusively associated with complete mineralization but also with the assimilation of organic carbon into cellular metabolism. Microalgae-based wastewater treatment has been described as a dual-function process, where complex organic substrates are simultaneously removed from the medium and converted into biomass, rather than being solely oxidized [54]. This metabolic coupling is particularly relevant in high-strength wastewater, where organic carbon serves as a substrate for mixotrophic growth.

##### Total Soluble Carbohydrate

**Figure 3:**
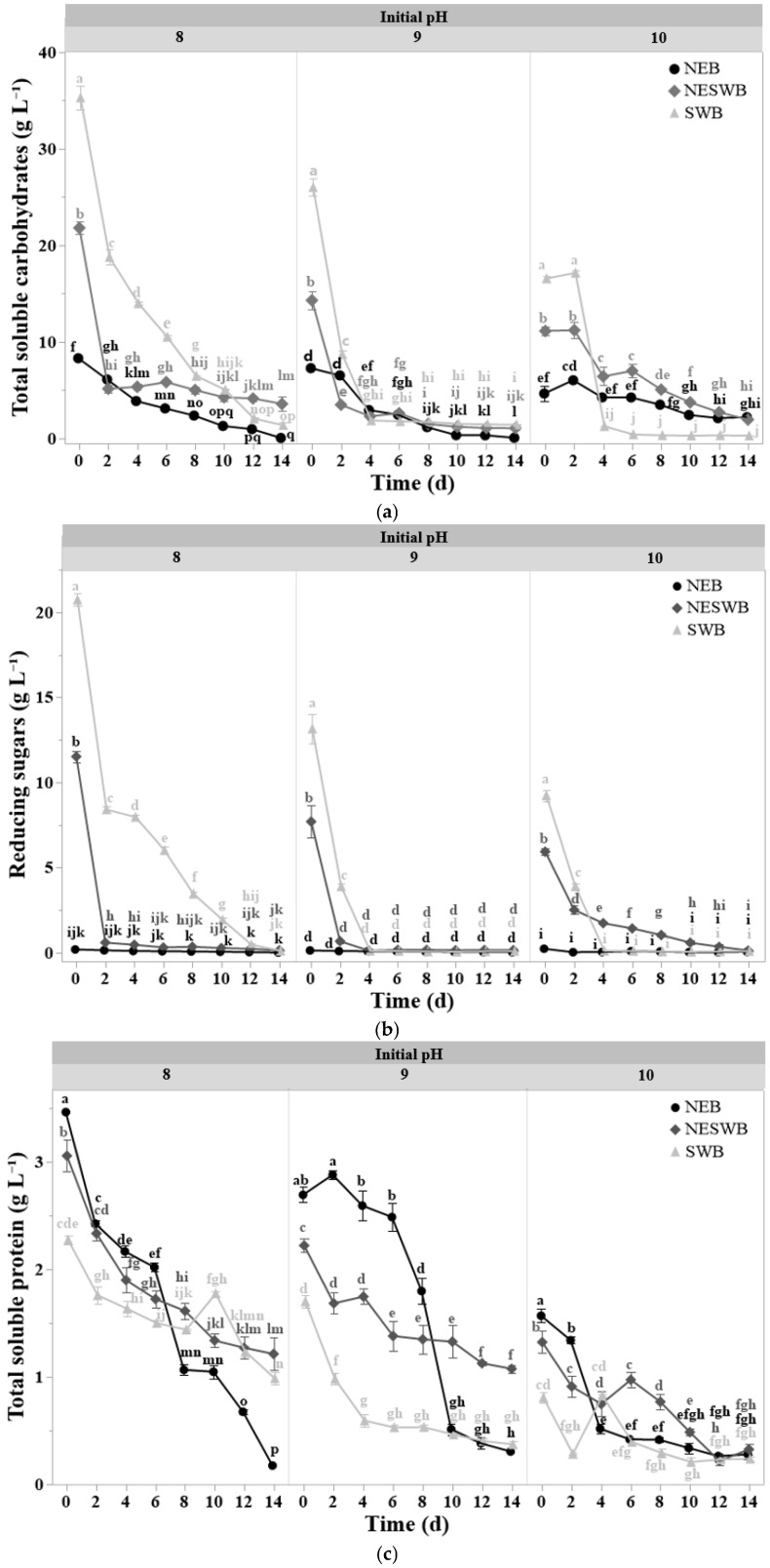
(**a**) Total soluble carbohydrates, (**b**) reducing sugar, and (**c**) total soluble protein contents in the supernatant during 14 days of phototrophic–mixotrophic cultivation of 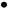 nejayote with biomass (NEB), 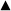 sweet whey with biomass (SWB), and 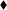 mixture of nejayote and sweet whey with biomass (NESWB), all inoculated with an alkaliphilic microalgae–cyanobacteria consortium (AMC) at initial pH 8, 9, or 10. Values are means of triplicate. Values in each panel without letters in common are significantly different (*p* < 0.05).

SWB exhibited an initial pH-dependent decrease in initial TSC, from 35.30 g L^−1^ at pH 8, 26.00 g L^−1^ at pH 9, and 16.60 g L^−1^ at pH 10 (Figure 3a). Previous reports showed carbohydrate concentrations ranging from 56.60 to 80.10 g L^−1^ in cheese whey [55]. The treatments NESWB and SWB at pH 8 exhibited average consumption rates during the 0–2 d interval (8.34 ± 0.37 and 8.26 ± 0.73 g L^−1^ day^−1^, respectively). By day 2, 77% of the initial TSC had been consumed in NESWB, whereas SWB reached 53%. Comparable rapid early uptake has been reported in mixotrophic systems supplied with mixed organic substrates, where multiple carbon forms stimulated broader metabolic engagement [12]. At pH 9, SWB exhibited a consumption rate of 8.65 ± 0.47 g L^−1^ d^−1^, reaching 67% of TSC consumption by 2 d. In contrast, in SWB at pH 10, TSC consumption started after 2 d. During the 2–4 d interval, the average consumption rate was 7.96 ± 0.11 g L^−1^ d^−1^, and by 4 d, 96% of the initial TSC had been consumed. This apparent delay likely reflects pH-driven changes in carbohydrate speciation, which temporarily change the analytically detectable soluble fraction before biological uptake predominates [56]. Previous studies have demonstrated that several microalgae and cyanobacteria can assimilate lactose via extracellular or cell-associated β-galactosidase activity, thereby converting lactose into glucose and galactose before cellular uptake [57,58]. This mechanism supports the observed high consumption rates in SWB and explains why TSC removal proceeded rapidly. In this context, the delayed but intense TSC depletion observed at pH 10 suggests an initial physiological adjustment phase, followed by activation of lactose hydrolysis and rapid carbon assimilation.

Control systems exhibited a moderate decrease in total soluble carbohydrates (TSC) over time, ranging from 17.9% to 24.4% depending on the initial pH (Table S1). For instance, in NESW at initial pH 10, TSC decreased from 26.24 to 19.83 g L^−1^, while at initial pH 8 and 9, similar reductions were observed. However, these changes followed a gradual and non-accelerated trend, indicating that they were not associated with biological consumption. The observed reduction in TSC in control systems can be attributed to physicochemical processes such as precipitation, complexation with divalent ions (Ca^2+^ present in nejayote), or partial transformation of soluble carbohydrates into less detectable forms under alkaline conditions.

##### Reducing Sugar

Rapid depletion of RSs was observed across all treatments (Figure 3b). Under mixotrophic conditions and light/dark cycling, microalgae can simultaneously harness inorganic carbon via photosynthesis during the light phase and organic carbon, such as monosaccharides and disaccharides, through heterotrophic pathways, thereby accelerating the consumption of readily metabolizable substrates. Light availability enhances ATP and NADPH generation, supporting photosynthetic carbon fixation and overall metabolic activity, while dark periods favor respiratory metabolism and continued organic carbon utilization, collectively resulting in increased reducing sugar depletion rates [59]. At pH 8, SWB exhibited the highest RS depletion rate (0–2 d: 6.16 ± 0.21 g L^−1^ d^−1^), followed by NESWB and then NE. SW is known to contain significant proportions of lactose (35–50 g L^−1^) [24], which microalgae and cyanobacteria can assimilate under mixotrophic conditions more rapidly than more complex carbohydrate matrices (xylose and arabinose).

At pH 9, NESWB exhibited the highest early reduction in reducing sugars during the first 2 d (3.50 ± 0.48 g L^−1^ d^−1^), whereas SWB and NE showed progressively lower rates. Conversely, at pH 10, SWB led the initial reducing sugar consumption (2.28 ± 0.08 g L^−1^d^−1^), followed by NESWB and NE. Sousa et al. [60] showed that reducing sugars serve as key additional carbon sources under mixotrophic growth, potentially enhancing biomass production and substrate uptake kinetics in *Chlorella vulgaris*.

Control systems exhibited a significant decrease in RSs over time across all culture media (NE, SW, and NESW) and initial pH conditions (8, 9, and 10) (Table S1). The magnitude of RSs depended on both factors. In general, reductions were observed under alkaline conditions (initial pH 9–10), with decreases of 40–43%, whereas at pH 8, there were reductions of 30–35%. Regarding the culture medium, SW and NESW showed RS decreases compared to NE. This indicated that the availability and type of soluble carbon sources strongly influenced the extent of transformation. The reduction in RSs in control systems suggests that reducing sugars are highly susceptible to abiotic transformations under alkaline conditions. These changes are likely associated with chemical degradation, isomerization reactions, or the formation of non-reducing compounds.

##### Total Soluble Protein

The initial TSP concentrations of NEB (1.56 ± 0.06–3.46 ± 0.01 g L^−1^) and SWB (0.80 ± 0.04–2.27 ± 0.04 g L^−1^) varied across initial pH conditions (8–10) (Figure 3c). Other reports on nejayote showed a TSP content of 2.23 g L^−1^ [61]. Protein depletion (0.27–0.52 g L^−1^ d^−1^) was observed at lower consumption rates than carbohydrates (8.26–8.65 g L^−1^ d^−1^), reflecting preferential utilization of readily available carbohydrate.

During phototrophic–mixotrophic cultivation at pH 8, NEB exhibited TSP depletion rates during the 0–2 d interval (0.52 ± 0.01 g L^−1^) and the 6–8 d interval (0.48 ± 0.03 g L^−1^ d^−1^), resulting in an overall protein consumption of 95.00 ± 2.80%. The biphasic depletion profile observed for NEB at pH 8 (0–2 and 6–8 d) suggests sequential availability and assimilation of soluble nitrogenous compounds throughout phototrophic–mixotrophic cultivation. NESWB showed depletion during the 0–2 d interval (0.36 ± 0.08 g L^−1^ d^−1^), followed by continued protein consumption, indicating continuous incorporation of soluble-protein-derived nitrogen during mixotrophic operation, whereas SWB showed TSP depletion during the 0–2 d interval (0.26 ± 0.05 g L^−1^ d^−1^), after which depletion rates decreased, suggesting that the remaining proteinaceous fraction may be less accessible. Consequently, the final TSP concentrations were 0.18 ± 0.01, 0.99 ± 0.06, and 1.21 ± 0.15 g L^−1^ for NEB, SWB, and NESWB, respectively.

At pH 9, NEB achieved the maximum consumption of TSP during 4 to 10 d of phototrophic–mixotrophic cultivation (0.35 ± 0.03 g L^−1^ d^−1^), while NESWB achieved consumption from 0 to 2 days (0.27 ± 0.06 g L^−1^ d^−1^) and SWB showed consumption from 0 to 2 days (0.28 ± 0.02 g L-1 d^−1^). The final protein concentrations were 0.31 ± 0.01, 0.37 ± 0.03, and 1.07 ± 0.04 g L^−1^ for NEB, SWB, and NESWB, respectively. Finally, at pH 10, all treatments showed similar consumption rates during the first 0–2 d (0.21–0.26 g L^−1^ d^−1^).

In SWB and NESWB, TSP concentrations increased on days 4 and 6, reaching 0.83 ± 0.01 and 0.97 ± 0.07 g L^−1^, respectively. These increases corresponded to protein production rates of 0.27 ± 0.01 g L^−1^ d^−1^ for SWB and 0.11 ± 0.07 g L^−1^ d^−1^ for NESWB. For the case of NE, Zayas-Olivares et al. [37] reported 7-day phototrophic–mixotrophic cultivation with a microalgae-cyanobacteria consortium in a bubble column photobioreactor operated under PAR 150 μmol m^−2^ s^−1^ with a 12/12 h light/dark cycle and without pH control, observing a decrease in soluble protein from 3.20 ± 0.10 to 2.00 ± 0.10 g L^−1^ (37.5%) in 7 days, with a decrease in pH from 11.10 ± 0.10 to 9.40 ± 0.20. Similarly, Del Valle-Real et al. [13] operated batch phototrophic–mixotrophic cultivation using nejayote as the sole substrate under alkaline conditions (initial pH ≈ 9.5), a 12:12 h light/dark cycle, and PAR intensities around 150 µmol m^−2^ s^−1^, reporting a decrease in soluble protein from approximately 10.0 to 2.0 mg mL^−1^ (≈ 80%) over 15 d. This behavior was consistent with that observed in mixotrophic microalgal systems cultivated in dairy-derived effluents. *C. vulgaris* cultivated on ricotta cheese whey at pH 6.5–7, a light intensity of 150 µmol m^−2^ s^−1^, and a temperature of 25.00 ± 2.00 °C achieved protein removal efficiencies of up to 55% for total nitrogen (TN) [62].

In contrast, the dynamics of total soluble protein (TSP) in the uninoculated control were strongly influenced by the composition of the culture medium and its interaction with the initial pH, rather than by pH as an independent factor (Table S1). At all time points, TSP values followed the order NE > NESW > SW, indicating that the substrate composition was the main determinant of soluble protein levels. Different temporal behaviors were observed among the treatments. In NE, TSP decreased gradually and consistently across all pH conditions. In NESW, TSP showed a markedly pH-dependent response, remaining relatively stable at initial pH values of 9 and 10, but decreasing markedly at an initial pH of 8, representing the greatest decrease among all control systems. In contrast, SW showed an initial decrease followed by a partial recovery toward the end of the culture period. At initial pH 8 and 9, SW showed slight net increases in TSP by day 14.

The observed TSP variations were likely due to the combined influence of physicochemical transformations and the metabolic activity of native microorganisms present in the substrates.

In whey-containing systems (SW and NESW), some of the observed behavior can be explained by the pH-dependent solubility of whey proteins. Major whey proteins, such as β-lactoglobulin and α-lactalbumin, exhibit minimum solubility near their isoelectric point (pH ~4.5–5.3), where aggregation and precipitation are favored [63]. In the present study, the rapid decrease in total soluble protein (TSP) observed in SW at an initial pH of 10 coincided with a marked decrease in the medium’s pH toward acidic values, suggesting that protein aggregation and phase redistribution contributed to the initial loss of soluble protein. The subsequent recovery of TSP indicates that these systems are dynamic, with reversible solubilization, redistribution between phases, or transformation of the protein fractions. In contrast, the more pronounced decrease in TSP observed in NESW at an initial pH of 8 suggests that protein behavior on mixed substrates is not governed solely by pH but also by interactions with the nejayote chemical matrix, particularly its high ionic strength and calcium content. Divalent ions, such as Ca^2+^, are known to promote aggregation, bridging, and protein complex formation, which can reduce protein solubility [64]. Therefore, the marked decrease in TSP in NESW likely reflects a combined effect of pH-induced solubility changes and ion-mediated interactions, rather than a single mechanism.

In NE, where protein concentrations were initially higher, the gradual decrease in TSP under all pH conditions suggests that protein solubility is progressively affected by the evolving physicochemical environment, including changes in ionic composition, interactions with organic matter, and possible adsorption to suspended solids.

In contrast, the present study demonstrates that controlling the initial pH and introducing a secondary organic substrate (SW) modifies not only the extent but also the temporal dynamics of soluble protein consumption.

##### Biomass Macromolecular Composition

At the beginning of phototrophic–mixotrophic cultivation, carbohydrate, protein, lipid, and ash contents were comparable across NEB, SWB, and NESWB, ranging from 15 to 16%, 21 to 23%, 20 to 23%, and 25 to 27% of dry biomass, respectively, with no significant differences among treatments. Najar-Almanzor et al. [65] reported carbohydrate and protein contents of 27–39% and 19–20%, respectively, during the initial stages of *H. pluvialis* cultivation in nejayote.

At day 14 (Figure 3a), the intracellular carbohydrate content in NEB increased with increasing initial pH (pH 8, 15.20 ± 1.80%; pH 9, 18.90 ± 1.20%; pH 10, 19.20 ± 1.20% dry weight), suggesting greater carbon fixation and storage under alkaline conditions, likely due to a relative limitation in nitrogen availability that favored the accumulation of carbon reserves. Conversely, a depletion was observed in SWB (pH 8, 13.70 ± 0.70%; pH 9, 10.90 ± 0.30%; pH 10, 5.50 ± 0.50% dry weight), indicating intensified metabolic utilization of carbon reserves under conditions of higher organic carbon and nitrogen availability. In contrast, NESWB showed no significant difference between pH levels. Intracellular carbohydrates are known to act as dynamic carbon reserves that are mobilized under nutrient depletion and metabolic stress, leading to carbon redistribution toward alternative metabolic pathways and endogenous metabolism [66,67].

Intracellular protein accumulation depended on initial pH (8, 9, and 10) and substrate composition (NE, SW, and NESW) (Figure 3b). The highest intracellular protein concentration was observed in NESWB at pH 10, reaching 30.50 ± 0.90% dry weight, compared with 21.90 ± 1.10% at the beginning of the phototrophic–mixotrophic cultivation. This behavior was consistent with previous studies showing that elevated pH (pH > 8.2) increased biosynthesis of structural cellular components, even when pH was imposed only as an initial condition [68]. Under alkaline pH conditions, microalgae can maintain intracellular pH homeostasis while benefiting from reduced microbial competition and efficient nutrient assimilation [69]. Supporting this trend at high pH, Zkeri et al. [70] cultivated *Chlorella sorokiniana* in dairy wastewater without pH adjustment, where the pH increased up to 10.45–10.84, and reported intracellular protein (54.6% dry weight) after 7-day batch experiments. Similarly, Sánchez-Zurano et al. [28] reported nitrogen recovery efficiencies exceeding 94% in *C. vulgaris* cultivated in whey (pH 7.50–8.10, 7 days), directly associated with greater intracellular protein accumulation. Likewise, Nazos et al. [46] demonstrated incorporation of whey-derived organic nitrogen into microalgal cellular protein during 7-day cultivations, supporting the role of dairy effluents in promoting protein-rich biomass.

**Figure 4:**
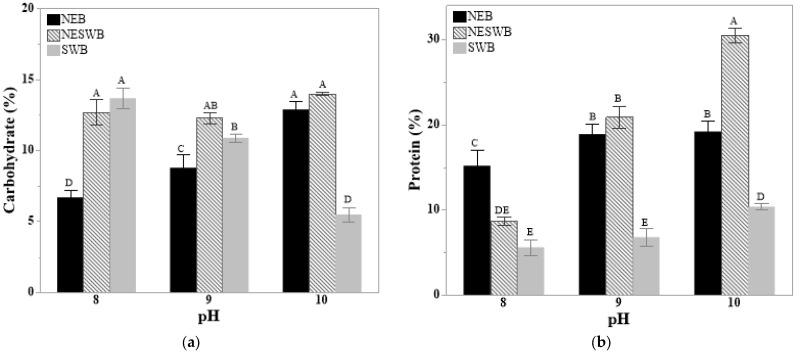

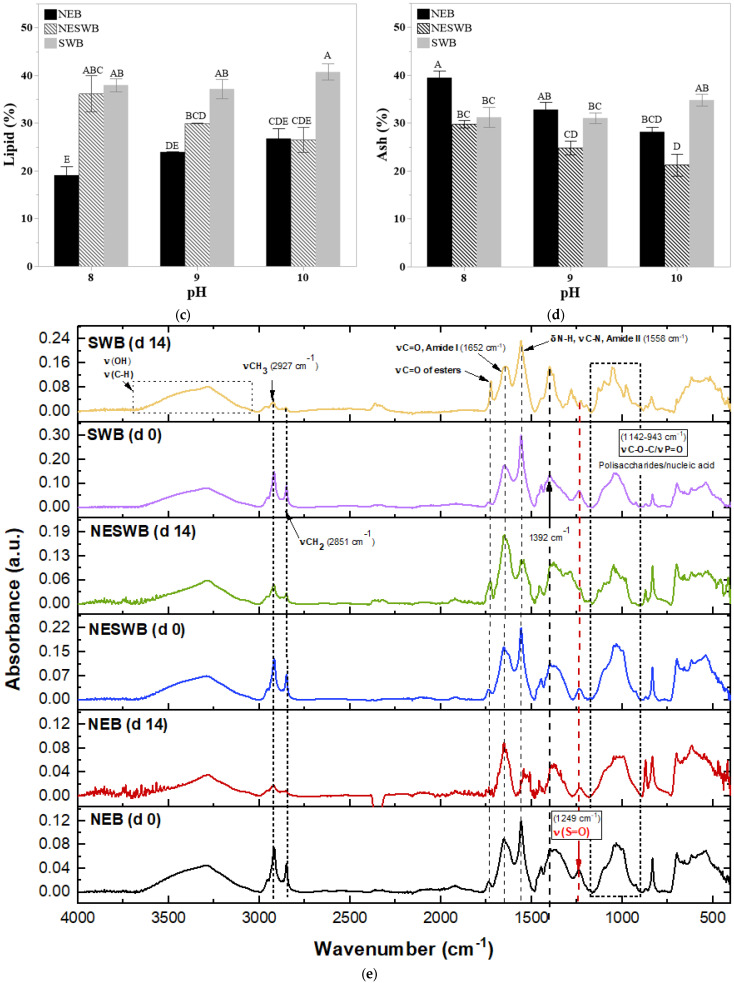
Intracellular components of biomass at day 14 from treatments, nejayote with biomass (NEB), sweet whey with biomass (SWB), and the mixture of nejayote and sweet whey with biomass (NESWB) at an initial pH of 8, 9, and 10. (**a**) Intracellular carbohydrate, (**b**) intracellular protein, (**c**) lipid, (**d**) ash, and (**e**) FTIR spectra of biomass collected at day 0 and day 14 from nejayote with biomass (NEB), sweet whey with biomass (SWB), and the mixture of nejayote and sweet whey with biomass (NESWB) at an initial pH of 10, cultivated with an alkaliphilic microalgae-cyanobacteria consortium (AMC). The spectra highlight major functional groups associated with cellular components. Values are means of triplicate. Values without letters in common are significantly different (*p* < 0.05).

The lipid content showed a contrasting response depending on the culture medium and initial pH (Figure 4c). In SWB, the lipid content was significantly higher than in the other treatments under all initial pH conditions, except for NESW with an initial pH of 8, ranging from 37.2 ± 3.6% to 40.7 ± 2.9% of dry weight, while the protein content remained significantly lower (5.6 ± 0.9–10.4 ± 0.4%). This behavior is consistent with previous studies showing that whey can act as an efficient source of organic carbon under mixotrophic culture, modifying the biochemical composition of the biomass according to the pH and nitrogen availability. In alkaliphilic microalgae, Youssef et al. [48] reported that whey-based culture under alkaline conditions altered the productivities of lipids, carbohydrates, and proteins, confirming that whey acts not only as a supplementary substrate but also as a metabolic driver of macromolecular partitioning. In NEB, the lipid content increased with increasing initial pH (no significant differences), rising from 19.1 ± 3.2% at pH 8 to 26.8 ± 3.6% at pH 10, while the protein content increased from 15.2 ± 1.8% to 19.2 ± 1.2%. Conversely, NESWB exhibited the opposite trend: the lipid content decreased (no significant differences) from 36.2 ± 6.6% at pH 8 to 26.6 ± 4.5% at pH 10, whereas the protein content increased significantly, reaching 30.5 ± 0.9% at pH 10. This inverse relationship suggests a redirection of carbon and nitrogen fluxes from storage lipids toward protein-enriched biomass under the combined substrate condition. Qu and Miao [71] demonstrated that alkaline pH can strongly alter carbon flux in microalgae; however, the direction of this redistribution depends on the physiological context. In *Chlorella* sp. BLD, alkaline conditions favored lipid accumulation at the expense of protein, whereas in the present study, the integrated substrate NESWB at an initial pH of 10 shifted the response toward protein accumulation. Alkaline conditions did not impose a universal lipid-rich phenotype but rather modulated biomass composition depending on the substrate context.

Ash content also showed significant differences between culture media and initial pH conditions (Figure 4d). In NEB, the ash content decreased significantly from 39.5 ± 2.4% at pH 8 to 28.2 ± 1.7% at an initial pH of 10, while in NESWB it decreased from 29.7 ± 1.4% to 21.3 ± 4.0%. In contrast, SWB showed no significant differences between pH conditions, remaining between 31.2 ± 3.7% and 34.8 ± 2.2%. These results are consistent with the mineral-rich nature of nejayote-based media, in which inorganic residues can contribute substantially to biomass ash [65]. The lower ash fraction in NESWB at pH 10, together with its higher protein content, suggests that the relative contribution of inorganic solids decreased as organic-biomass-associated fractions increased under this condition. In wastewater-grown algal biomass, elevated ash is common and often substantially higher than in cleaner cultivation systems. Previous studies have reported that wastewater algal biomass may contain around 30–50 wt% ash, and high-rate algal pond biomass around 15–30 wt% ash [72], which agrees with the relatively high ash fractions observed here, particularly in NEB and SWB.

##### ATR-FTIR Spectral Characterization of Biomass

The FTIR spectra revealed marked differences in the band intensity, appearance, and relative distribution of functional groups of biomass obtained from six samples—NEB, NESWB, and SWB on days 0 and 14 (Figure 4e). In all samples, a broad band between 3200 and 3500 cm^−1^ was observed, corresponding to O-H and N-H stretching vibrations [73]. This band was more intense at 14 d in the SWB sample. This increase suggests a greater contribution from hydroxylated compounds and hydrogen bonds, which are commonly associated with the polysaccharide and protein components of EPSs (exopolysaccharides). The region between 2927 and 2850 cm^−1^, assigned to the asymmetric and symmetric stretching of CH_3_ and CH_2_ groups, was present in all spectra. A decrease in intensity was observed on day 14 in NEB, SWB, and NESWB, suggesting a relative decrease in aliphatic chains associated with lipid consumption. A distinctive band near 1730–1740 cm^−1^, attributed to the C=O stretching of ester groups, was weak in the NEB samples on day 0. This band increased in NESWB and SWB on day 14, suggesting the formation of ester-containing compounds, such as lipids or EPS-related esters, during phototrophic–mixotrophic cultivation. The amide I band, at approximately 1652 cm^−1^, was present in all spectra (NEB, SWB, and NESWB; days 0 and 14) but showed notable variations. In the NEB samples, the band intensity decreased. In contrast, NESWB showed an increase on day 14 (28%).

The mathematical deconvolution of the amide I region (1600–1700 cm^−1^) revealed significant changes in the relative distribution of protein secondary structures as a function of phototrophic–mixotrophic cultivation time and culture medium (Table 3). Across all treatments, α-helix content decreased significantly from day 0 to day 14, while β-turn structures increased significantly. A significant increase in β-sheet content at day 14 (37.70 ± 2.20%) and a significant reduction in random coil structures were observed in SWB. This behavior suggests a more ordered and compact protein organization [74]. In NEB and NESWB, α-helix content declined from ~34–35% to 23–25%, whereas β-turn contributions increased to 34.00 ± 1.70% and 31.90 ± 2.60%, respectively. The loss of α-helices and the enrichment of β-turns, without pronounced β-sheet formation, indicate a structural adaptation to the highly alkaline and ionic environment rather than tight protein packing. These results indicate that substrate composition modulates not only the amount of protein but also its structural organization within the biomass.

##### SDS-PAGE Profiling of Intracellular Proteins

**Figure 5:**
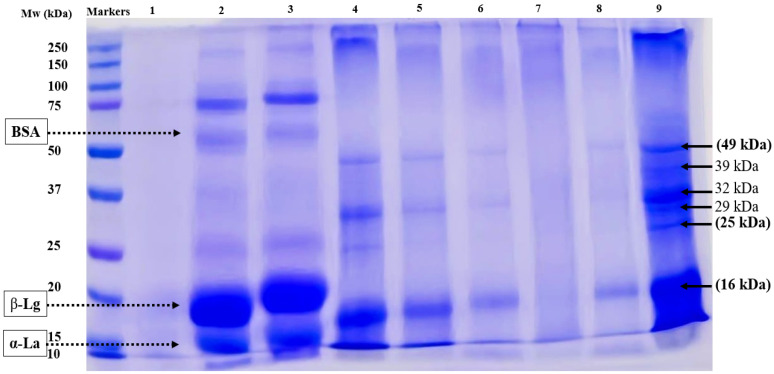
SDS-PAGE protein profiles of nejayote (NE in line 1), sweet whey (SW in line 2) and a combination of nejayote and sweet whey (NESW in line 3) fermented with alkaliphilic microorganisms consortia at pH 10, demonstrating the changes in intracellular biomass proteins at day 0 (NEB in line 4, SWB in line 5 and NESWB in line 6) and after 14 d of phototrophic–mixotrophic cultivation (NEB in line 7, SWB in line 8 and NESWB in line 9). The molecular weights (kDa) of protein standard (markers) are indicated on the left side of the gel. Bands corresponding to identified or tentatively assigned proteins are indicated in parentheses, whereas other bands are reported based on their apparent molecular weight only.

The intracellular protein composition of AMC biomass varied with the culture medium and phototrophic-mixotrophic cultivation time (Figure 5). As expected, SWB showed intense bands of bovine serum albumin (BSA), β-lactoglobulin (β-Lg) and α-lactalbumin (α-La), and their intensity was reduced in NESWB. For nejayote proteins, the band intensity was low and not well defined, likely due to the predominance of low-molecular-weight proteins and peptides (<10 kDa) as previously reported by Buitimea-Cantúa et al. [75]. Then, the biomass recovered immediately after the inoculation (day 0) in these three treatments differed in the intensity of the bands (45 and 32 kDa), indicating that intracellular proteins of the AMC were released into the culture broth. This intracellular protein release was more pronounced in NESWB (absence of signal at 25 kDa) on day 0. However, for day 14, the band intensity increased, and bands appeared (25, 39, and 29 kDa). Notably, NESWB biomass at day 14 exhibited multiple well-defined bands distributed approximately between 16 and 45 kDa. This molecular weight range was consistent with previous reports on intracellular protein extracts from microalgae, in which most metabolic, structural, and enzymatic proteins are detected between 10 and 80 kDa [76]. Cutshaw et al. [77] analyzed *Chlorella sorokiniana* biomass and identified several intracellular proteins with molecular weights comparable to those observed in this study, including phosphoglycerate kinase (49 kDa), 50S ribosomal protein L7/L12 (chloroplast) (16 kDa), and Fe-superoxide dismutase (26 kDa). In such studies, an increase in the number and intensity of SDS-PAGE bands has been associated with elevated protein biosynthesis rather than with extracellular contamination. Furthermore, the absence of intense bands corresponding to whey-derived proteins, such as β-lactoglobulin (~18 kDa) and α-lactalbumin (~14 kDa) [78], in the day 14 biomass lanes suggests that extracellular whey proteins were not merely adsorbed onto the cell surface but were hydrolyzed and metabolized, supporting active assimilation rather than passive accumulation. Similar conclusions have been reported in studies focused on intracellular protein extraction from microalgae, where extracellular proteins are absent from biomass profiles when active metabolic uptake and intracellular synthesis occur [76].

The increase in intracellular protein bands observed by SDS-PAGE correlates with FTIR results, which showed an increase in the amide I band and modifications in the amide II region at 14 d. These changes are indicative of variations in protein-related functional groups and band profiles, suggesting alterations in the biochemical composition of the biomass. However, it should be emphasized that SDS-PAGE provides qualitative information on protein profiles, while FTIR reflects changes in functional groups within the whole biomass rather than direct quantification of protein content or biosynthetic activity. Similar associations between FTIR amide bands and changes in protein-related composition have been reported in microalgal systems cultivated on complex organic substrates. Irshad et al. [79] described similar correlations between FTIR amide bands and increased accumulation of intracellular proteins during mixotrophic microalgal culture on complex organic substrates. These authors used food waste hydrolysates; as a result, their microalgal cultures showed increased protein-associated FTIR signals, reflecting the redirection of assimilated carbon and nitrogen toward intracellular protein synthesis rather than carbohydrate storage. Similarly, Parakh & Tong [80] conducted studies combining tofu whey with food waste digestate, demonstrating that the availability of diverse organic carbon and nitrogen sources promotes metabolic allocation toward protein-rich biomass, resulting in higher intracellular protein content and improved single-cell protein production. Taken together, these results demonstrate that under alkaline conditions and in the presence of mixed substrates, the consortium redirected assimilated carbon and nitrogen toward intracellular protein synthesis rather than toward extracellular accumulation or carbohydrate storage.

## 4. Conclusions

This study demonstrates that the strategic combination of SW and NE creates a synergistic substrate that enhances the performance of an alkaliphilic microalgae–cyanobacteria consortium. Compared to single-residue treatments, NESWB promoted greater TSS accumulation, reaching up to 7.01 ± 0.25 g L^−1^ at initial pH 9. The use of a metabolically diverse consortium was key to this performance. The functional complementarity among consortium members enabled rapid carbohydrate consumption, with up to 77% TSC depletion within the first 2 days, sustained COD removal, and stable biomass formation under alkaline conditions. Operation at alkaline initial pH (initial pH 9–10) ensured stable biological pH regulation via photosynthetic proton uptake, thereby maintaining the culture pH within the alkaline range throughout cultivation. The highest intracellular protein content was observed with the combined substrate at initial pH 10, reaching 30.50 ± 0.90% (dry weight), compared with 19.2 % and 10.4 % for SW and NE, respectively. Under these conditions, COD removal efficiency reached 91.66% in NEB and exceeded 70% in NESW treatments. These characteristics demonstrate the potential of combining these highly pollutant substrates to grow alkaliphilic microalgae–cyanobacteria consortia as a promising platform for sustainable biorefineries based on agro-industrial waste in Mexico.

## Figures and Tables

**Table 1:** Initial characterization of nejayote (NE), whey (SW), and nejayote–whey (NESW) wastewater.

Parameter	Nejayote (NE)	Whey (SW)	Nejayote–Whey (NESW)
pH	10.62 ± 0.08 ^A^	5.65 ± 0.06 ^C^	9.20 ± 0.08 ^B^
DO (%)	28.30 ± 1.40 ^A^	32.20 ± 4.20 ^A^	31.40 ± 2.30 ^A^
COD (g L^−1^)	18.40 ± 0.17 ^C^	71.00 ± 0.25 ^A^	56.10 ± 0.08 ^B^
ORP (mV)	–256.17 ± 14.60 ^C^	42.50 ± 2.45 ^A^	–68.60 ± 23.30 ^B^
Conductivity (mS cm^−1^)	6.50 ± 0.07 ^A^	5.62 ± 0.06 ^C^	5.84 ± 0.04 ^B^
TSS (g L^−1^)	0.69 ± 0.16 ^C^	16.97 ± 2.11 ^A^	9.98 ± 0.18 ^B^

Values are means of triplicate. Values in each row without letters in common are significantly different (*p* < 0.05).

**Table 2:** Total phosphorus (TP) and total nitrogen (TN) removal efficiency (%) at selected cultivation times (2, 8, and 14 d) for nejayote with biomass (NEB), sweet whey with biomass (SWB), and the mixture of nejayote and sweet whey with biomass (NESWB) at initial pH values of 8, 9, and 10, cultivated with an alkaliphilic microalgae–cyanobacteria consortium.

Initial pH	Time (d)	TP Removal Efficiency (%)			TN Removal Efficiency (%)		
		NEB	SWB	NESWB	NEB	SWB	NESWB
8	2	46.33 ± 29.43 ^BCD^	3.56 ± 5.31 ^FGH^	24.33 ± 14.50 ^CDEFG^	73.78 ± 10.68 ^C^	10.78 ± 4.67 ^IJK^	23.00 ± 8.71 ^GH^
	8	59.67 ± 21.92 ^B^	11.00 ± 7.63 ^EFG^	26.44 ± 17.20 ^CDEFG^	72.44 ± 13.45 ^C^	17.67 ± 3.52 ^HIJ^	36.11 ± 10.31 ^F^
	14	90.89 ± 4.42 ^A^	22.22 ± 9.57 ^CDEFG^	47.00 ± 6.33 ^BCD^	93.56 ± 2.22 ^A^	23.22 ± 3.96 ^GH^	70.00 ± 7.84 ^CD^
9	2	20.56 ± 15.66 ^DEFG^	15.11 ± 10.36 ^EFG^	24.33 ± 18.20 ^CDEFG^	32.67 ± 10.87 ^FG^	60.33 ± 6.48 ^DE^	37.89 ± 15.22 ^F^
	8	54.22 ± 4.52 ^B^	25.67 ± 13.08 ^CDEFG^	26.44 ± 17.20 ^CEFG^	53.44 ± 10.99 ^E^	78.6 ± 7.60 ^BC^	72.67 ± 6.86 ^C^
	14	47.78 ± 10.87 ^BC^	35 ± 16.49 ^BCDE^	47.00 ± 6.33 ^BCD^	72.44 ± 6.26 ^C^	87.33 ± 3.93 ^AB^	91.56 ± 3.16 ^A^
10	2	–16.89 ± 18.22 ^H^	10.56 ± 11.88 ^EFFG^	–23.22 ± 24.49 ^H^	7.33 ± 4.98 ^JK^	87.89 ± 5.07 ^AB^	56.11 ± 1.80 ^E^
	8	2.56 ± 17.94 ^FGH^	15.11 ± 3.98 ^EFG^	12.22 ± 9.19 ^EFG^	10.00 ± 2.17 ^IJK^	92.33 ± 2.84 ^A^	92.56 ± 3.35 ^A^
	14	27.22 ± 5.16 ^CDEF^	18.67 ± 3.91 ^EFG^	36.67 ± 6.18 ^BCDE^	18.78 ± 4.32 ^HI^	94.00 ± 1.69 ^A^	96.44 ± 1.03 ^A^

Values are means of triplicate. Values in each row without letters in common are significantly different (*p* < 0.05).

**Table 3:** Relative distribution of protein secondary structures (%) in intracellular biomass. Biomass collected at day 0 and day 14 from nejayote with biomass (NEB), sweet whey with biomass (SWB), and the mixture of nejayote and sweet whey with biomass (NESWB) at an initial pH of 10, cultivated with an alkaliphilic microalgae–cyanobacteria consortium (AMC).

Secondary Structure	NEB		NESWB		SWB	
	Day 0	Day 14	Day 0	Day 14	Day 0	Day 14
β-sheet	28.10 ± 0.30 ^B^	31.40 ± 1.50 ^B^	28.40 ± 0.80 ^B^	31.60 ± 1.50 ^B^	30.70 ± 3.70 ^B^	37.70 ± 2.20 ^A^
Random	13.90 ± 0.60 ^B^	11.00 ± 1.40 ^BC^	13.90 ± 0.20 ^B^	11.90 ± 1.30 ^BC^	17.00 ± 0.80 ^A^	10.90 ± 1.50 ^C^
α-Helix	34.70 ± 0.30 ^A^	23.60 ± 1.60 ^B^	34.30 ± 1.20 ^A^	24.60 ± 0.50 ^B^	32.60 ± 2.60 ^A^	24.10 ± 2.10 ^B^
β-Turn	23.30 ± 0.60 ^CD^	34.00 ± 1.70 ^A^	23.50 ± 0.60 ^CD^	31.90 ± 2.60 ^AB^	19.60 ± 1.60 ^D^	27.30 ± 2.60 ^BC^

Values are means of triplicate. Values in each row without letters in common are significantly different (*p* < 0.05).

## Data Availability

The original contributions presented in the study are included in the article/Supplementary Material; further inquiries can be directed to the corresponding author.

## Supplementary Materials

The following supporting information can be downloaded at: https://www.mdpi.com/article/10.3390/foods15112022/s1. Figure S1: Time-course profiles of pH during bioreaction kinetics over 14 days of cultivation of nejayote with biomass (NEB), sweet whey with biomass (SWB), and a mixture of nejayote and sweet whey with biomass (NESWB) inoculated with an alkaliphilic microalgae-cyanobacteria consortium (AMC). Data are grouped by initial pH (8, 9, and 10). Figure S2: Time-course profiles of oxygen demand (OD) during bioreaction kinetics over 14 days of cultivation of nejayote with biomass (NEB), sweet whey with biomass (SWB), and a mixture of nejayote and sweet whey with biomass (NESWB) inoculated with an alkaliphilic microalgae–cyanobacteria consortium (AMC). Data are grouped by initial pH (8, 9, and 10). Table S1: Physicochemical parameters of uninoculated control systems (NE, SW, NESW) at different initial pH values (8, 9, and 10) during 14 days of cultivation.

## Abbreviations

The following abbreviations are used in this manuscript:

AMC	Alkaliphilic microalgae–cyanobacteria consortium
ATR	Attenuated total reflection
COD	Chemical oxygen demand
DO	Dissolved oxygen
EPSs	Exopolysaccharides
FTIR	Fourier transform infrared spectroscopy
MSM	Mineral salt medium
NE	Nejayote (maize lime-cooking wastewater)
NEB	Nejayote with biomass
NESW	Nejayote–sweet whey mixture
NESWB	Nejayote–sweet whey mixture with biomass
ORP	Oxidation–reduction potential
PAR	Photosynthetically active radiation
RSs	Reducing sugars
SDS-PAGE	Sodium dodecyl sulfate–polyacrylamide gel electrophoresis
SW	Sweet whey
SWB	Sweet whey with biomass
TSC	Total soluble carbohydrates
TSP	Total soluble proteins
TSS	Total suspended solids

## References

[1] Díaz-Montes E., Castro-Muñoz R. (2022). Analyzing the Phenolic Enriched Fractions from Nixtamalization Wastewater (Nejayote) Fractionated in a Three-Step Membrane Process. Curr. Res. Food Sci..

[2] Valenzuela E.I., Gutiérrez-Uribe J.A., Franco-Morgado M., Cervantes-Avilés P. (2024). Navigating the Waters of Nixtamalization: Sustainable Solutions for Maize-Processing Wastewater Treatment. Sci. Total Environ..

[3] Najar-Almanzor C.E., González-Díaz R.L., García-Cayuela T., Carrillo-Nieves D. (2025). Adaptation and Bioremediation Efficiency of UV-Mutagenized Microalgae in Undiluted Agro-Industrial Effluents from Mexico. Environments.

[4] Salah A., Sany H., El-Sayed A.E.-K.B., El-Bahbohy R.M., Mohamed H.I., Amin A. (2023). Growth Performance and Biochemical Composition of *Desmodesmus* sp. Green Alga Grown on Agricultural Industries Waste (Cheese Whey). Water Air Soil Pollut..

[5] Selmi H., Presutto E., Spano G., Capozzi V., Fragasso M. (2025). Valorising Whey: From Environmental Burden to Bio-Based Production of Value-Added Compounds and Food Ingredients. Foods.

[6] Uribe-Velázquez T., Díaz-Vázquez D., Barajas-Álvarez P., González-López M.E., Gradilla-Hernández M.S., Garcia-Amezquita L.E., Carrillo-Nieves D., García-Cayuela T. (2025). From Waste to Value: Mitigating the Environmental Impact of Whey in Jalisco, Mexico. J. Clean. Prod..

[7] Ramírez-Jiménez A.K., Castro-Muñoz R. (2021). Emerging Techniques Assisting Nixtamalization Products and By-Products Processing: An Overview. Crit. Rev. Food Sci. Nutr..

[8] Zayas T., De Gante A., Arvide M.G.T., Hernández M.V., Soriano-Moro G., Salgado L. (2022). Treatment of Nixtamalization Wastewater (Nejayote) Using Electrocoagulation and Combined Chemical Coagulation/Electrocoagulation Processes. Desalination Water Treat..

[9] López-Pacheco I.Y., Carrillo-Nieves D., Salinas-Salazar C., Silva-Núñez A., Arévalo-Gallegos A., Barceló D., Afewerki S., Iqbal H.M.N., Parra-Saldívar R. (2019). Combination of Nejayote and Swine Wastewater as a Medium for *Arthrospira Maxima* and *Chlorella vulgaris* Production and Wastewater Treatment. Sci. Total Environ..

[10] Daneshvar E., Sik Ok Y., Tavakoli S., Sarkar B., Shaheen S.M., Hong H., Luo Y., Rinklebe J., Song H., Bhatnagar A. (2021). Insights into Upstream Processing of Microalgae: A Review. Bioresour. Technol..

[11] Fal S., Smouni A., Arroussi H.E. (2023). Integrated Microalgae-Based Biorefinery for Wastewater Treatment, Industrial CO_2_ Sequestration and Microalgal Biomass Valorization: A Circular Bioeconomy Approach. Environ. Adv..

[12] Garza-Valverde E., García-Gómez C., Nápoles-Armenta J., Samaniego-Moreno L., Martínez-Orozco E., Mora-Orozco C.D.L. (2024). Nejayote and Food Waste Leachate as a Medium for *Scenedesmus acutus* and *Haematococcus pluvialis* Production: A Mixture Experimental Design. Water.

[13] Del Valle-Real M., Franco-Morgado M., García-García R., Guardado-Félix D., Gutiérrez-Uribe J.A. (2023). Wastewater from Maize Lime-Cooking as Growth Media for Alkaliphilic Microalgae–Cyanobacteria Consortium to Reduce Chemical Oxygen Demand and Produce Biomass with High Protein Content. Int. J. Food Sci. Technol..

[14] Wang S., Liu J., Li C., Chung B.M. (2019). Efficiency of *Nannochloropsis oculata* and *Bacillus polymyxa* Symbiotic Composite at Ammonium and Phosphate Removal from Synthetic Wastewater. Environ. Technol..

[15] Ouhsassi M., Khay E.O., Bouyahya A., El Ouahrani A., Harsal A.E., Abrini J. (2020). Evaluation of Self-Purifying Power of Cyanobacteria *Pseudanabaena galeata*: Case of Dairy Factory Effluents. Appl. Water Sci..

[16] Kedves A., Kónya Z. (2025). Enhancing Granule Formation: The Role of Chitosan and Chitosan Nanoparticles in Microalgal-Bacterial Granular Sludge Development. Environ. Technol. Innov..

[17] Kant Bhatia S., Ahuja V., Chandel N., Mehariya S., Kumar P., Vinayak V., Saratale G.D., Raj T., Kim S.-H., Yang Y.-H. (2022). An Overview on Microalgal-Bacterial Granular Consortia for Resource Recovery and Wastewater Treatment. Bioresour. Technol..

[18] Elystia S., Effendi I., Amri A., Efriyeldi (2025). Development of Granular Indigenous Microalgal-Bacterial Consortium (G-IMBC) Technology for Palm Oil Mill Effluent Treatment: Optimization, Characterization, and Microbial Community Dynamics. Environ. Chall..

[19] Tang C.-C., Hu Y.-R., He Z.-W., Li Z.-H., Tian Y., Wang X.C. (2024). Promoting Symbiotic Relationship between Microalgae and Bacteria in Wastewater Treatment Processes: Technic Comparison, Microbial Analysis, and Future Perspectives. Chem. Eng. J..

[20] Sukačová K., Červený J. (2017). Can Algal Biotechnology Bring Effective Solution for Closing the Phosphorus Cycle? Use of Algae for Nutrient Removal—Review of Past Trends and Future Perspectives in the Context of Nutrient Recovery. Eur. J. Environ. Sci..

[21] Ekpan F.M., Ori M.O., Samuel H.S. (2025). Effects of Environmental Factors and Nutrient Availability on the Biochemical Composition of Algae for Biofuel Production. Discov. Appl. Sci..

[22] Abdelfattah A., Ali S.S., Ramadan H., El-Aswar E.I., Eltawab R., Ho S.-H., Elsamahy T., Li S., El-Sheekh M.M., Schagerl M. (2023). Microalgae-Based Wastewater Treatment: Mechanisms, Challenges, Recent Advances, and Future Prospects. Environ. Sci. Ecotechnol..

[23] Bhat O., Unpaprom Y., Ramaraj R. (2023). *Spirulina* Cultivation Under Different Light-Emitting Diodes for Boosting Biomass and Protein Production. Mol. Biotechnol..

[24] Pires A.F., Marnotes N.G., Rubio O.D., Garcia A.C., Pereira C.D. (2021). Dairy By-Products: A Review on the Valorization of Whey and Second Cheese Whey. Foods.

[25] Granada-Moreno C.I., Aburto-Medina A., de los Cobos Vasconcelos D., González-Sánchez A. (2017). Microalgae Community Shifts during the Biogas Upgrading in an Alkaline Open Photobioreactor. J. Appl. Microbiol..

[26] de los Cobos-Vasconcelos D., García-Cruz E.L., Franco-Morgado M., González-Sánchez A. (2016). Short-Term Evaluation of the Photosynthetic Activity of an Alkaliphilic Microalgae Consortium in a Novel Tubular Closed Photobioreactor. J. Appl. Phycol..

[27] Laura L.B., American Public Health Association, American Water Works Association, Water Environment Federation (2017). Standard Methods for the Examination of Water and Wastewater.

[28] O’Dell J.W., Environmental Monitoring Systems Laboratory (1996). Method 410.4—The Determination of Chemical Oxygen Demand by Semi-Automated Colorimetry. Methods for the Determination of Metals in Environmental Samples.

[29] DuBois M., Gilles K.A., Hamilton J.K., Rebers P.A., Smith F. (1956). Colorimetric Method for Determination of Sugars and Related Substances. Anal. Chem..

[30] Lowry O.H., Rosebrough N.J., Farr A.L., Randall R.J. (1951). Protein Measurement with the Folin Phenol Reagent. J. Biol. Chem..

[31] Miller G.L. (1959). Use of dinitrosaIicyIic Acid Reagent for Determination of Reducing Sugar. Anal. Chem..

[32] Pruvost J., Van Vooren G., Le Gouic B., Couzinet-Mossion A., Legrand J. (2011). Systematic Investigation of Biomass and Lipid Productivity by Microalgae in Photobioreactors for Biodiesel Application. Bioresour. Technol..

[33] Sirés I., Brillas E. (2012). Remediation of Water Pollution Caused by Pharmaceutical Residues Based on Electrochemical Separation and Degradation Technologies: A Review. Environ. Int..

[34] Lu Q., Zhou W., Min M., Ma X., Ma Y., Chen P., Zheng H., Doan Y.T.T., Liu H., Chen C. (2016). Mitigating Ammonia Nitrogen Deficiency in Dairy Wastewaters for Algae Cultivation. Bioresour. Technol..

[35] Amiri M., Hosseini S.E., Asadi G., Khayambashi B., Abedinia A. (2024). Optimization of Microalgae Protein Extraction from *Scenedesmus obliquus* and Investigating Its Functional Properties. LWT.

[36] Román-Escobedo L.C., Cristiani-Urbina E., Morales-Barrera L. (2024). Bioremediation with an Alkali-Tolerant Yeast of Wastewater (Nejayote) Derived from the Nixtamalization of Maize. Fermentation.

[37] Sánchez-Zurano A., Villaró-Cos S., Ciardi M., Acién-Fernández F.G., Fernández-Sevilla J.M., Lafarga T. (2024). Assessment of the Mixotrophic Production of *Chlorella vulgaris* Using Milk Whey as a Nutrient Source. J. Appl. Phycol..

[38] Çoklar H., Akbulut M., Aygun A., Akbulut M.T. (2025). Valorization of Dairy By-Products, Sweet Whey, and Acid Whey, in the Production of Fermented Black Carrot Juice: A Comparative Study of the Phytochemical, Physicochemical, Microbiological, and Sensorial Aspects. Foods.

[39] Zheng Y., Yu X., Li T., Xiong X., Chen S. (2014). Induction of D-Xylose Uptake and Expression of NAD(P)H-Linked Xylose Reductase and NADP + -Linked Xylitol Dehydrogenase in the Oleaginous Microalga *Chlorella sorokiniana*. Biotechnol. Biofuels.

[40] Rubiyatno, Matsui T., Mori K., Toyama T. (2021). Paramylon Production by *Euglena gracilis* via Mixotrophic Cultivation Using Sewage Effluent and Waste Organic Compounds. Bioresour. Technol. Rep..

[41] Xu H., Liu C., Wang A., Yue B., Lin T., Ding M. (2024). Microalgae Treatment of Food Processing Wastewater for Simultaneous Biomass Resource Recycling and Water Reuse. J. Environ. Manag..

[42] Wu M., Wu G., Lu F., Wang H., Lei A., Wang J. (2022). Microalgal Photoautotrophic Growth Induces pH Decrease in the Aquatic Environment by Acidic Metabolites Secretion. Biotechnol. Biofuels Bioprod..

[43] Abiusi F., Wijffels R.H., Janssen M. (2020). Doubling of Microalgae Productivity by Oxygen Balanced Mixotrophy. ACS Sustain. Chem. Eng..

[44] Cunha W.R., Cottas A.G., Teixeira T.A., de Ferreira J.S. (2020). Evaluation of Phicocyanin Production by *Anabaena variabilis* Using Different Organic Carbon Sources. J. Eng. Exact. Sci..

[45] Labbé J.I., Ramos-Suárez J.L., Hernández-Pérez A., Baeza A., Hansen F. (2017). Microalgae Growth in Polluted Effluents from the Dairy Industry for Biomass Production and Phytoremediation. J. Environ. Chem. Eng..

[46] Khalaji M., Hosseini S.A., Ghorbani R., Agh N., Rezaei H., Kornaros M., Koutra E. (2023). Treatment of Dairy Wastewater by Microalgae *Chlorella vulgaris* for Biofuels Production. Biomass Convers. Biorefin..

[47] Stratigakis N.C., Nazos T.T., Goumenaki M., Tsolakidi A., Spantidaki M., Lagouvardou-Spantidaki A., Ghanotakis D.F. (2025). Growth Performance and Adaptability of an EPS-Producing *Chlorella* Strain in Cheese Whey with High and Low Salinity: Prospects for the Sustainable Production of Microalgal Biomass. J. Appl. Phycol..

[48] Youssef A.M., Gomaa M., Mohamed A.K.S.H., El-Shanawany A.-R.A. (2024). Enhancement of Biomass Productivity and Biochemical Composition of Alkaliphilic Microalgae by Mixotrophic Cultivation Using Cheese Whey for Biofuel Production. Environ. Sci. Pollut. Res..

[49] Zayas-Olivares A., Pérez-Cortés M., Franco-Morgado M., Montilla A., López-Revenga P., Gutiérrez-Uribe J.A. (2026). Coupled Organic Matter Degradation and Dynamic Extracellular Polysaccharide Turnover by an Alkaline Microalgae–Cyanobacteria Consortium Treating Maize Lime Cooking Wastewater. J. Environ. Manag..

[50] Paulenco A., Vintila A.C.N., Vlaicu A., Ciltea-Udrescu M., Galan A.-M. (2023). *Nannochloris* sp. Microalgae Strain for Treatment of Dairy Wastewaters. Microorganisms.

[51] Valizadeh K., Davarpanah A. (2020). Design and Construction of a Micro-Photo Bioreactor in Order to Dairy Wastewater Treatment by Micro-Algae: Parametric Study. Energy Sources Part Recovery Util. Environ. Eff..

[52] Caporgno M.P., Taleb A., Olkiewicz M., Font J., Pruvost J., Legrand J., Bengoa C. (2015). Microalgae Cultivation in Urban Wastewater: Nutrient Removal and Biomass Production for Biodiesel and Methane. Algal Res..

[53] Secretaría de Gobernación Norma Oficial Mexicana NOM-001SEMARNAT-2021, Que Establece los Límites Permisibles de Contaminantes en las Descargas de Aguas Residuales en Cuerpos Receptores Propiedad de la Nación. https://www.dof.gob.mx/nota_detalle.php?codigo=5645374&fecha=11/03/2022#gsc.tab=0.

[54] Macaluso M., Chiellini C., Ciurli A., Guglielminetti L., Najar B., Taglieri I., Sanmartin C., Bianchi A., Venturi F., Zinnai A. (2022). Application of Five Different *Chlorella* sp. Microalgal Strains for the Treatment of Vegetation Waters Derived from Unconventional Oil Extractions Enriched with Citrus Byproducts. Foods.

[55] Nazos T.T., Stratigakis N.C., Spantidaki M., Lagouvardou Spantidaki A., Ghanotakis D.F. (2023). Characterization of Cheese Whey Effluents and Investigation of Their Potential to Be Used as a Nutrient Substrate for *Chlorella* Biomass Production. Waste Biomass Valorization.

[56] Wei H., Zhang S., Cheng P., Hu P. (2025). Unraveling the behaviors of extracellular polymeric substances for microalgae harvesting through starch-based flocculants via repeated adjustments in pH. J. Environ. Chem. Eng..

[57] Li Y., Miros S., Kiani H., Eckhardt H.-G., Blanco A., Mulcahy S., McDonnell H., Tiwari B.K., Halim R. (2023). Mechanism of Lactose Assimilation in Microalgae for the Bioremediation of Dairy Processing Side-Streams and Co-Production of Valuable Food Products. J. Appl. Phycol..

[58] Bentahar J., Doyen A., Beaulieu L., Deschênes J.-S. (2019). Investigation of β-Galactosidase Production by Microalga *Tetradesmus obliquus* in Determined Growth Conditions. J. Appl. Phycol..

[59] Thepsuthammarat K., Reungsang A., Plangklang P. (2023). Microalga *Coelastrella* sp. Cultivation on Unhydrolyzed Molasses-Based Medium towards the Optimization of Conditions for Growth and Biomass Production under Mixotrophic Cultivation. Molecules.

[60] Sousa A.C., Dias C., Martins A.R., Gomes A.G., Santos C.A. (2025). Using Winery Effluents for Cultivating Microalgae as Bio-Additives for Vineyards. J. Appl. Phycol..

[61] Contreras-Jácquez V., Valenzuela-Vázquez U., Grajales-Hernández D.A., Mateos-Díaz J.C., Arrellano-Plaza M., Jara-Marini M.E., Asaff-Torres A. (2022). Pilot-Scale Integrated Membrane System for the Separation and Concentration of Compounds of Industrial Interest from Tortilla Industry Wastewater (Nejayote). Waste Biomass Valorization.

[62] Casá N.E., Lois-Milevicich J., Alvarez P., Mateucci R., De Escalada Pla M. (2022). *Chlorella vulgaris* Cultivation Using Ricotta Cheese Whey as Substrate for Biomass Production. J. Appl. Phycol..

[63] Pelegrine D.H.G., Gasparetto C.A. (2005). Whey Proteins Solubility as Function of Temperature and pH. LWT—Food Sci. Technol..

[64] Jorge N., Santos C., Teixeira A.R., Marchão L., Tavares P.B., Lucas M.S., Peres J.A. (2022). Treatment of Agro-Industrial Wastewaters by Coagulation-Flocculation-Decantation and Advanced Oxidation Processes—A Literature Review. Eng. Proc..

[65] Najar-Almanzor C.E., García-Cayuela T., Gutierrez-Uribe J.A., Carrillo-Nieves D. (2026). Bioremediation of Alkaline Corn Wastewater with *Haematococcus pluvialis* under Laboratory and 100 L Raceway Pond Conditions. Sci. Rep..

[66] Chen H.-H., Jiang J.-G. (2017). Lipid Accumulation Mechanisms in Auto-and Heterotrophic Microalgae. J. Agric. Food Chem..

[67] Kato Y., Oyama T., Inokuma K., Vavricka C.J., Matsuda M., Hidese R., Satoh K., Oono Y., Chang J.-S., Hasunuma T. (2021). Enhancing Carbohydrate Repartitioning into Lipid and Carotenoid by Disruption of Microalgae Starch Debranching Enzyme. Commun. Biol..

[68] Vadlamani A., Viamajala S., Pendyala B., Varanasi S. (2017). Cultivation of Microalgae at Extreme Alkaline pH Conditions: A Novel Approach for Biofuel Production. ACS Sustain. Chem. Eng..

[69] Jin D., Zhang X., Zhou L., Zhang X., Wu P. (2024). Emerging Applications and Mechanisms of Algal-Bacterial Symbiosis on Sustainable Wastewater Treatment: A Comprehensive Review. J. Water Process Eng..

[70] Zkeri E., Iliopoulou A., Katsara A., Korda A., Aloupi M., Gatidou G., Fountoulakis M.S., Stasinakis A.S. (2021). Comparing the Use of a Two-Stage MBBR System with a Methanogenic MBBR Coupled with a Microalgae Reactor for Medium-Strength Dairy Wastewater Treatment. Bioresour. Technol..

[71] Qu D., Miao X. (2021). Carbon Flow Conversion Induces Alkali Resistance and Lipid Accumulation under Alkaline Conditions Based on Transcriptome Analysis in *Chlorella* sp. BLD. Chemosphere.

[72] Molino A., Iovine A., Casella P., Mehariya S., Chianese S., Cerbone A., Rimauro J., Musmarra D. (2018). Microalgae Characterization for Consolidated and New Application in Human Food, Animal Feed and Nutraceuticals. Int. J. Environ. Res. Public Health.

[73] Parra-Riofrío G., García-Márquez J., Casas-Arrojo V., Uribe-Tapia E., Abdala-Díaz R.T. (2020). Antioxidant and Cytotoxic Effects on Tumor Cells of Exopolysaccharides from *Tetraselmis suecica* (Kylin) Butcher Grown Under Autotrophic and Heterotrophic Conditions. Mar. Drugs.

[74] Qoms M.S., Wong S.K., Fauzi N.M., Husain K., Makpol S., Tan J.K. (2025). Microalgae-Derived Peptides Targeting Lifestyle-Related Diseases: Discovery, Mechanisms, Structure–Activity Relationships, and Structural Modifications. Antioxidants.

[75] Buitimea-Cantúa N.E., Antunes-Ricardo M., Gutiérrez-Uribe J.A., Del Refugio Rocha-Pizaña M., De La Rosa-Millán J., Torres-Chávez P.I. (2020). Protein-Phenolic Aggregates with Anti-Inflammatory Activity Recovered from Maize Nixtamalization Wastewaters (Nejayote). LWT.

[76] Anjos L., Estêvão J., Infante C., Mantecón L., Power D.M. (2022). Extracting Protein from Microalgae (*Tetraselmis chuii*) for Proteome Analysis. MethodsX.

[77] Cutshaw A., Frost H., Uludag-Demirer S., Liu Y., Liao W. (2023). Protein Extraction, Precipitation, and Recovery from *Chlorella sorokiniana* Using Mechanochemical Methods. Energies.

[78] Khazdooz L., Zarei A., Meletharayil G., Kapoor R., Abbaspourrad A. (2023). Synthesis of a Cation-Exchange Resin by Inverse Suspension Polymerization for Lactoferrin Extraction from Whey. ACS Omega.

[79] Irshad S., Tang T., Nawaz A., Zu Y., Yuan Z., Li Y., Liao X., Li Z., Wang M., Ni L. (2025). Utilizing Food Waste Hydrolysate for Enhanced Microalgae Protein Production: A Step towards Circular Economy. J. Clean. Prod..

[80] Parakh S.K., Tong Y.W. (2025). Co-Utilizing Tofu Whey with Food Waste Digestate Enhances Techno-Economic Feasibility of Microalgal Single-Cell Protein Production. Bioresour. Technol..

