# Supplementary material for: Use of an Alkaline Wastewater Stream to Increase the Initial pH of Whey and Recover a Microbial Biomass with High Protein Content
Source: Foods. 2026 Jun 4;15(11):2022. doi: 10.3390/foods15112022 (PMC13256802; doi:10.3390/foods15112022)
Supplement: Supplementary file 1 [file foods-15-02022-s001.zip › foods-4237459-supplementary.pdf]

(Supplementary material)

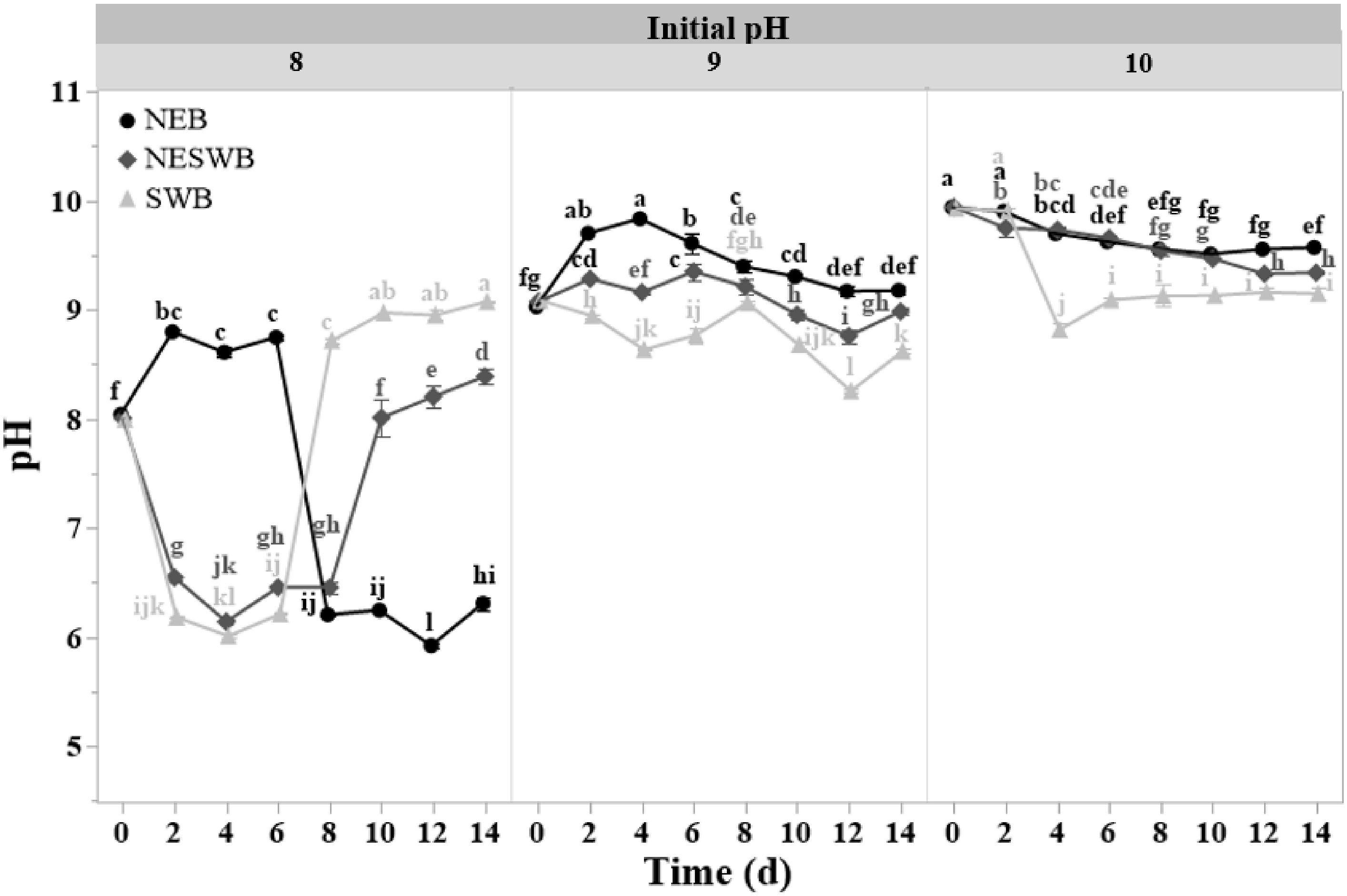

Figure S1. Time-course profiles of pH during phototrophic-mixotrophic cultivation over 14 days of cultivation of nejayote with biomass (NEB), sweet whey with biomass (SWB), and mixture of nejayote-sweet whey with biomass (NESWB) inoculated with an alkaliphilic microalgae–cyanobacteria consortium (AMC). Data are grouped by initial pH (8, 9, and 10).

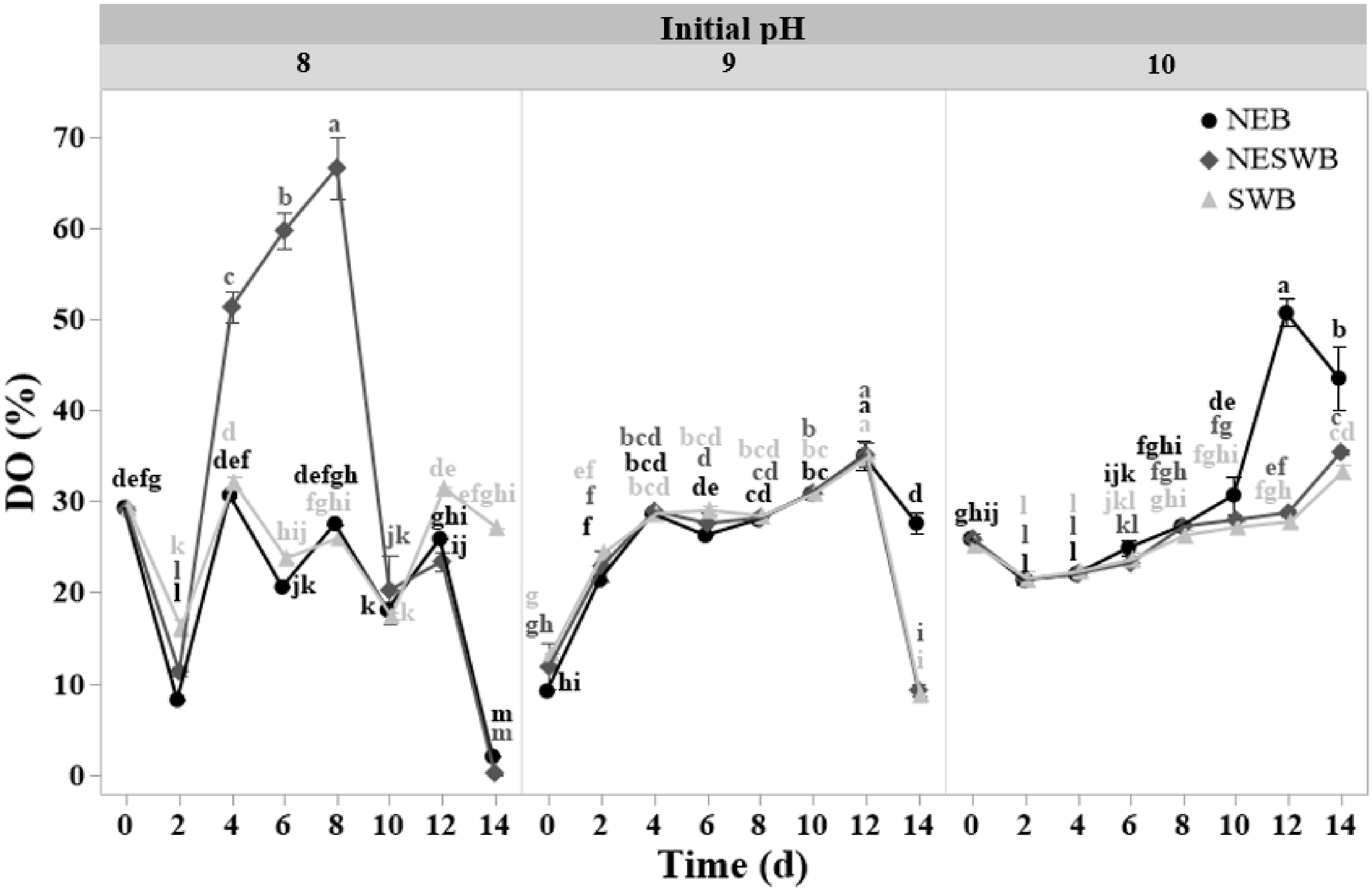

Figure S2. Time-course profiles of oxygen demand (OD) during phototrophic-mixotrophic cultivation over 14 days of cultivation of nejayote with biomass (NEB), sweet whey with biomass (SWB), and mixture of nejayote-sweet whey with biomass (NESWB) inoculated with an alkaliphilic microalgae–cyanobacteria consortium (AMC). Data are grouped by initial pH (8, 9, and 10)

Table S1. Physicochemical parameters of uninoculated control systems (NE, SW, NESW) at different initial pH values (8, 9, and 10) during 14 days of cultivation.

Time (d)	Culture media	Initial pH	Average COD (g L^{-1})	SD COD (g L^{-1})	Average TSC (g L^{-1})	SD TSC (g L^{-1})	Average RS (g L^{-1})	SD RS (g L^{-1})	Average TSP (g L^{-1})	SD TSP (g L^{-1})	Average TSS (g L^{-1})	SD TSS (g L^{-1})	Average OD (%)	SD OD (%)	Average pH	SD pH
**0**	**NESW**	**10**	35.27	1.89	26.24	1.05	17.01	0.73	3.68	0.32	7.11	1.33	7.83	0.25	9.88	0.04
**2**	**NESW**	**10**	30.67	0.67	24.73	1.89	12.70	0.64	3.82	0.23	5.24	0.53	0.00	0.00	6.40	0.02
**4**	**NESW**	**10**	27.43	0.21	20.17	0.31	10.69	0.51	3.85	0.14	5.84	0.80	0.00	0.00	5.29	0.02
**6**	**NESW**	**10**	28.57	0.15	21.27	0.85	9.45	0.38	3.90	0.07	6.52	1.05	0.00	0.00	5.73	0.02
**8**	**NESW**	**10**	25.10	0.89	20.83	0.40	9.62	0.36	3.95	0.09	6.29	0.62	0.00	0.00	6.54	0.01
**10**	**NESW**	**10**	31.37	0.64	21.23	1.16	9.50	0.64	3.66	0.07	6.35	0.17	0.00	0.00	6.81	0.02
**12**	**NESW**	**10**	32.53	0.09	20.43	0.68	8.65	0.50	3.45	0.16	6.25	1.01	0.00	0.00	7.22	0.02
**14**	**NESW**	**10**	32.93	0.08	19.83	1.30	9.76	0.52	3.58	0.07	6.36	0.44	0.00	0.00	7.13	0.03
**0**	**NESW**	**9**	42.97	2.15	35.67	0.96	24.22	1.82	3.39	0.18	7.42	0.49	7.87	0.25	9.01	0.01
**2**	**NESW**	**9**	53.17	2.25	35.70	0.56	24.45	1.49	3.49	0.09	7.61	0.51	0.00	0.00	5.21	0.01
**4**	**NESW**	**9**	52.50	2.15	33.60	0.98	22.29	2.01	3.35	0.05	6.83	0.50	0.00	0.00	4.88	0.01
**6**	**NESW**	**9**	47.90	2.31	31.23	0.87	19.33	0.31	3.48	0.06	6.42	0.15	0.00	0.00	5.05	0.01
**8**	**NESW**	**9**	45.47	2.61	31.27	1.27	18.81	0.75	3.57	0.12	6.55	0.34	0.00	0.00	5.34	0.04
**10**	**NESW**	**9**	41.40	0.26	29.53	0.64	16.66	0.50	3.50	0.12	6.62	0.28	0.00	0.00	5.77	0.02
**12**	**NESW**	**9**	41.77	3.65	29.73	1.62	16.57	1.32	3.42	0.18	6.82	0.44	0.00	0.00	6.09	0.01
**14**	**NESW**	**9**	37.07	4.10	29.27	0.57	14.28	1.06	3.51	0.11	7.12	0.13	0.00	0.00	6.03	0.02
**0**	**NESW**	**8**	50.43	1.82	44.43	0.80	34.51	2.09	3.60	0.12	7.56	0.06	7.90	0.66	7.94	0.01
**2**	**NESW**	**8**	51.13	1.22	44.93	0.40	33.99	1.63	3.41	0.15	4.92	0.11	0.00	0.00	5.14	0.02
**4**	**NESW**	**8**	53.00	2.16	46.57	0.31	29.42	1.19	3.21	0.04	5.85	0.33	0.00	0.00	4.82	0.02
**6**	**NESW**	**8**	43.63	2.63	43.67	1.10	30.30	1.18	3.26	0.01	7.27	0.10	0.00	0.00	5.65	0.03
**8**	**NESW**	**8**	40.27	1.46	39.63	1.42	28.35	1.00	2.80	0.20	8.75	0.30	0.00	0.00	6.41	0.01
**10**	**NESW**	**8**	36.53	2.75	38.73	1.20	28.73	0.38	3.09	0.09	9.11	0.33	0.00	0.00	6.42	0.00
**12**	**NESW**	**8**	38.87	2.66	33.50	1.30	23.69	1.43	2.61	0.05	10.76	0.28	0.00	0.00	6.46	0.01
**14**	**NESW**	**8**	41.27	1.34	33.97	1.57	22.76	2.12	2.45	0.16	11.28	0.11	0.00	0.00	7.15	0.03
**0**	**NE**	**10**	16.62	0.53	11.93	0.47	4.15	0.19	4.35	0.19	4.26	0.20	9.37	0.21	9.93	0.01

**2**	**NE**	**10**	16.57	0.31	10.36	0.59	3.61	0.36	4.29	0.12	3.80	0.08	0.00	0.00	8.08	0.02
**4**	**NE**	**10**	16.30	0.62	10.70	0.49	4.34	0.97	4.39	0.07	4.00	0.03	0.00	0.00	8.14	0.01
**6**	**NE**	**10**	16.50	0.36	10.16	0.58	3.56	0.46	4.34	0.07	4.08	0.00	0.00	0.00	8.36	0.02
**8**	**NE**	**10**	15.83	0.15	10.64	0.07	3.22	0.24	4.19	0.23	4.37	0.16	0.00	0.00	8.40	0.01
**10**	**NE**	**10**	16.03	0.96	9.72	1.23	2.36	0.30	4.26	0.11	4.46	0.51	0.00	0.00	8.41	0.01
**12**	**NE**	**10**	16.00	0.26	8.38	1.16	2.09	0.20	4.01	0.02	4.89	0.21	0.00	0.00	8.49	0.01
**14**	**NE**	**10**	15.37	1.27	10.77	0.74	2.62	0.15	3.92	0.18	5.15	0.07	0.00	0.00	8.34	0.02
**0**	**NE**	**9**	23.13	2.11	14.73	0.50	6.53	0.64	4.32	0.14	3.39	0.21	11.43	0.57	8.97	0.02
**2**	**NE**	**9**	24.37	1.42	13.63	0.45	5.76	0.68	4.26	0.05	3.19	0.21	0.00	0.00	8.11	0.07
**4**	**NE**	**9**	20.20	1.31	15.13	0.21	4.97	0.73	4.16	0.21	3.85	0.04	0.00	0.00	8.08	0.01
**6**	**NE**	**9**	19.13	0.25	14.30	0.46	5.06	0.91	4.03	0.14	3.73	0.16	0.00	0.00	8.55	0.02
**8**	**NE**	**9**	18.27	0.75	13.13	0.35	5.00	0.37	4.09	0.12	3.90	0.00	0.00	0.00	8.25	0.04
**10**	**NE**	**9**	18.77	0.65	13.17	0.35	5.32	0.66	3.88	0.09	3.90	0.25	0.00	0.00	8.15	0.01
**14**	**NE**	**9**	14.23	2.36	15.13	0.21	6.46	0.17	3.85	0.12	3.90	0.00	0.00	0.00	7.84	0.04
**0**	**NE**	**8**	30.20	1.85	21.37	0.86	10.55	0.87	4.06	0.12	3.93	0.30	4.83	0.21	7.99	0.02
**2**	**NE**	**8**	29.50	1.15	23.27	0.76	10.80	1.09	4.35	0.35	2.10	0.06	0.00	0.00	7.84	0.01
**4**	**NE**	**8**	23.57	2.44	21.17	0.90	9.69	0.59	4.31	0.07	1.86	0.31	0.00	0.00	7.81	0.02
**6**	**NE**	**8**	26.10	0.30	19.90	0.40	8.91	0.51	4.06	0.08	3.06	0.03	0.00	0.00	8.28	0.01
**8**	**NE**	**8**	23.73	0.95	19.17	0.35	6.89	0.49	4.11	0.02	2.80	0.06	0.00	0.00	7.99	0.01
**10**	**NE**	**8**	19.33	0.68	17.47	1.42	8.14	0.39	3.89	0.22	3.64	0.06	0.00	0.00	7.82	0.01
**12**	**NE**	**8**	19.47	0.60	18.40	1.55	7.70	1.52	3.80	0.15	3.51	0.07	0.00	0.00	7.73	0.01
**14**	**NE**	**8**	16.57	0.76	15.87	0.93	6.36	1.00	3.79	0.12	3.27	0.64	0.93	0.51	7.92	0.02
**0**	**SW**	**10**	55.99	3.11	41.67	1.46	31.44	0.86	3.63	0.26	4.13	0.24	22.30	0.46	9.96	0.06
**2**	**SW**	**10**	64.44	0.11	45.63	1.15	34.24	1.51	2.30	0.04	13.25	0.06	0.00	0.00	9.14	0.02
**4**	**SW**	**10**	71.88	0.41	46.63	0.96	36.30	0.97	2.25	0.06	10.28	0.09	0.00	0.00	5.05	0.04
**6**	**SW**	**10**	44.88	0.28	39.07	0.38	27.13	1.57	2.52	0.15	10.13	0.20	0.00	0.00	5.30	0.01
**8**	**SW**	**10**	47.61	0.39	38.87	1.91	24.24	0.82	2.25	0.21	9.94	0.64	0.00	0.00	4.84	0.02
**10**	**SW**	**10**	40.83	0.44	34.70	1.85	24.10	0.51	2.51	0.20	9.70	0.60	0.00	0.00	4.92	0.02
**12**	**SW**	**10**	36.21	0.62	30.93	1.40	24.47	0.84	2.51	0.05	10.12	0.39	0.00	0.00	5.08	0.02

**14**	**SW**	**10**	35.65	0.20	36.73	1.80	27.35	1.73	2.74	0.07	11.36	1.00	0.00	0.00	4.93	0.01
**0**	**SW**	**9**	58.33	0.02	49.53	0.70	38.75	0.20	2.75	0.11	12.13	0.38	24.50	0.61	8.96	0.05
**2**	**SW**	**9**	70.80	0.80	51.70	0.85	38.87	1.56	2.42	0.17	12.33	0.30	0.00	0.00	5.54	0.02
**4**	**SW**	**9**	69.85	0.12	50.37	1.97	36.04	0.62	2.56	0.09	12.20	0.71	0.00	0.00	4.74	0.02
**6**	**SW**	**9**	60.63	2.20	46.97	2.63	33.09	1.13	2.58	0.05	9.22	0.57	0.00	0.00	4.97	0.02
**8**	**SW**	**9**	52.97	2.50	43.33	1.72	33.82	0.77	2.60	0.14	10.61	0.35	0.00	0.00	5.13	0.01
**10**	**SW**	**9**	45.77	1.00	46.87	0.55	34.72	0.52	2.54	0.18	12.34	0.44	0.00	0.00	5.52	0.01
**12**	**SW**	**9**	44.47	3.63	42.40	1.66	31.42	1.93	2.73	0.14	12.73	0.25	0.00	0.00	5.96	0.01
**14**	**SW**	**9**	41.10	1.05	41.50	2.14	31.33	1.00	2.82	0.17	11.78	0.68	0.00	0.00	5.86	0.02
**0**	**SW**	**8**	69.27	0.81	58.50	2.69	49.32	1.00	2.85	0.05	13.70	0.29	28.20	1.87	7.93	0.02
**2**	**SW**	**8**	63.23	2.24	46.63	3.75	34.95	2.45	2.28	0.21	14.04	0.33	12.63	0.74	4.86	0.02
**4**	**SW**	**8**	57.83	0.95	42.97	2.26	34.31	1.96	2.83	0.21	14.25	0.09	0.00	0.00	4.58	0.02
**6**	**SW**	**8**	57.13	1.46	45.00	0.75	31.53	1.04	2.65	0.09	15.13	0.40	0.00	0.00	5.32	0.01
**8**	**SW**	**8**	51.80	1.82	39.93	0.35	29.74	0.91	2.90	0.02	15.84	0.47	0.00	0.00	6.18	0.02
**10**	**SW**	**8**	55.17	2.12	37.17	4.05	27.57	0.71	2.66	0.01	15.40	0.85	0.00	0.00	6.21	0.01
**12**	**SW**	**8**	46.30	3.68	36.20	1.00	28.24	0.76	2.74	0.06	15.17	0.48	0.00	0.00	6.26	0.01
**14**	**SW**	**8**	43.20	2.23	35.47	1.23	23.87	1.49	2.98	0.20	14.39	0.21	0.00	0.00	6.14	0.02
